# Potential effects of a high CO_2_ future on leguminous species

**DOI:** 10.1002/pei3.10009

**Published:** 2020-04-24

**Authors:** Stacy D. Singer, Syama Chatterton, Raju Y. Soolanayakanahally, Udaya Subedi, Guanqun Chen, Surya N. Acharya

**Affiliations:** ^1^ Agriculture and Agri‐Food Canada Lethbridge Research and Development Centre Lethbridge AB Canada; ^2^ Agriculture and Agri‐Food Canada Saskatoon Research and Development Centre Saskatoon SK Canada; ^3^ Department of Agricultural, Food and Nutritional Science University of Alberta Edmonton AB Canada

**Keywords:** climate change, forage, photosynthesis, pulse, quality, stress resilience, yield

## Abstract

Legumes provide an important source of food and feed due to their high protein levels and many health benefits, and also impart environmental and agronomic advantages as a consequence of their ability to fix nitrogen through their symbiotic relationship with rhizobia. As a result of our growing population, the demand for products derived from legumes will likely expand considerably in coming years. Since there is little scope for increasing production area, improving the productivity of such crops in the face of climate change will be essential. While a growing number of studies have assessed the effects of climate change on legume yield, there is a paucity of information regarding the direct impact of elevated CO_2_ concentration (e[CO_2_]) itself, which is a main driver of climate change and has a substantial physiological effect on plants. In this review, we discuss current knowledge regarding the influence of e[CO_2_] on the photosynthetic process, as well as biomass production, seed yield, quality, and stress tolerance in legumes, and examine how these responses differ from those observed in non‐nodulating plants. Although these relationships are proving to be extremely complex, mounting evidence suggests that under limiting conditions, overall declines in many of these parameters could ensue. While further research will be required to unravel precise mechanisms underlying e[CO_2_] responses of legumes, it is clear that integrating such knowledge into legume breeding programs will be indispensable for achieving yield gains by harnessing the potential positive effects, and minimizing the detrimental impacts, of CO_2_ in the future.

## Graphical abstract

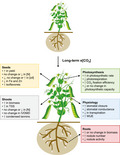

## 1 INTRODUCTION

The Leguminosae family comprises approximately 800 genera and 20,000 species, and constitutes the third largest family of flowering plants (Stagnari et al., 2017). In terms of agronomically important crops, grain legumes (pulses), soybean, and certain forage species (e.g., alfalfa [*Medicago sativa*]) provide value as both food and feed due to their high levels of protein and health‐promoting properties (Darmadi‐Blackberry et al., 2004; Graham & Vance, 2003; Singh et al., 2017). In addition, their capacity to fix nitrogen through their symbiotic relationship with rhizobia allows approximately 50–70 Mt of nitrogen to be fixed annually in agricultural systems (Herridge, Peoples, & Boddey, 2008). This reduces the need for synthetic nitrogen fertilizers, which currently support 30%–50% of non‐leguminous crop yields (Erisman et al., 2008), with more than 50% of applied fertilizer typically lost from cereal crops as runoff or to the atmosphere in the form of the highly potent greenhouse gas, nitrous oxide (N_2_O; Ladha, 2016; Stagnari et al., 2017). Furthermore, the production of synthetic ammonia presently consumes 1.5% of total global primary energy (US Energy Information Administration, 2015) and generates further N_2_O and carbon dioxide (CO_2_) as by‐products (Foyer et al., 2016). Therefore, decreasing our use of such fertilizers would minimize the environmental footprint of crop production considerably.

Due to their health, cropping and environmental benefits, as well as the fact that our global population is expected to reach upwards of 9.8 billion by 2050 and 11.2 billion by 2100 (United Nations, 2017), the demand for leguminous crops will almost certainly escalate substantially in coming years. Although small overall global increases in their production have been observed over the last half century, with the exception of soybean, these have been achieved mainly through expansions of cropping area (Foyer et al., 2016), which suggests that the rate of genetic yield enhancement in these crops has been relatively low. Considering that the majority of arable land is already being utilized for crop production, and that even less may be available in the future as a result of factors such as urbanization, salinization, and desertification (Alexandratos & Bruinsma, 2012), it is imperative that efforts are made to harness the substantial untapped potential present in legumes for yield improvements.

Unfortunately, the climate‐related changes that are predicted to occur within this century will lend further challenges to meeting demand in the future. Rising levels of anthropogenic greenhouse gases are leading to global warming, with 1°C increases already evident compared to pre‐industrial times (Allen et al., 2018) and up to 4°C increases possible by the end of the century under certain emission scenarios (Collins et al., 2013). Escalating temperatures also bring about other downstream climatic effects, such as increases in the severity and incidence of droughts, floods, and other extreme weather events (Allen et al., 2018), which all have an impact on legume production. While small increases in temperature may have positive effects in the short term, at least in certain temperate regions, increasingly high temperatures and drought will eventually reduce biomass and seed production, as well as quality (Myers et al., 2014). Indeed, negative effects on legume production arising from climate change are already apparent in many regions of the world. For example, soybean (*Glycine max*) yields in the United States have declined by 2%–4% for every 1°C increase in temperature over the growing season between 1994 and 2013, which has led to losses of US$11 billion (Mourtzinis et al., 2015).

While much attention is being paid to the impact of greenhouse gas emissions on crop productivity in the context of these secondary climate change effects, less importance has been placed on the direct consequences of increased greenhouse gas levels. The rapid rise in atmospheric CO_2_ concentration ([CO_2_]) that is occurring due to a variety of anthropogenic factors, including our continued reliance of fossil fuels and deforestation, is a major driving force of climate change (Solomon et al., 2009). Currently, levels of this greenhouse gas have already increased more than 40% since pre‐industrial times and now exceed 400 µmol/mol (Mauna Loa Observatory measurement as of June 5, 2019). This is the highest that CO_2_ levels have been at any time in the past 650,000 years and possibly the last 23 million years (Intergovernmental Panel on Climate Change (IPCC), 2007). Unfortunately, concentrations are projected to continue to escalate between approximately 440 and 550 µmol/mol by 2050 and could reach as high as approximately 900 µmol/mol by the end of the century (Meinshausen et al., 2011).

Since CO_2_ acts as the primary substrate for photosynthesis, which is critical for crop growth, such a large alteration in its atmospheric levels will have direct implications on all agricultural systems (Tausz et al., 2013). There is also growing evidence to suggest that alterations in [CO_2_] can have a substantial impact on various other physiological and developmental processes in plants, which could exacerbate consequences for future production. However, due to the distinct physiology of legumes, these effects may be somewhat atypical compared to other plant species. In this review, we will discuss the potential impacts of elevated [CO_2_] (e[CO_2_]) on legumes in the context of photosynthesis, yield, quality, and ability to withstand various types of stress. Advancing our knowledge in this area and furthering downstream research to clarify these effects will be of the utmost importance for the development of new, high‐yielding, climate‐smart cultivars as a contribution to food security in the future.

## 2 EFFECT OF ELEVATED [CO_2_] ON PHOTOSYNTHESIS AND BIOMASS PRODUCTION IN LEGUMES

**Figure 1:**
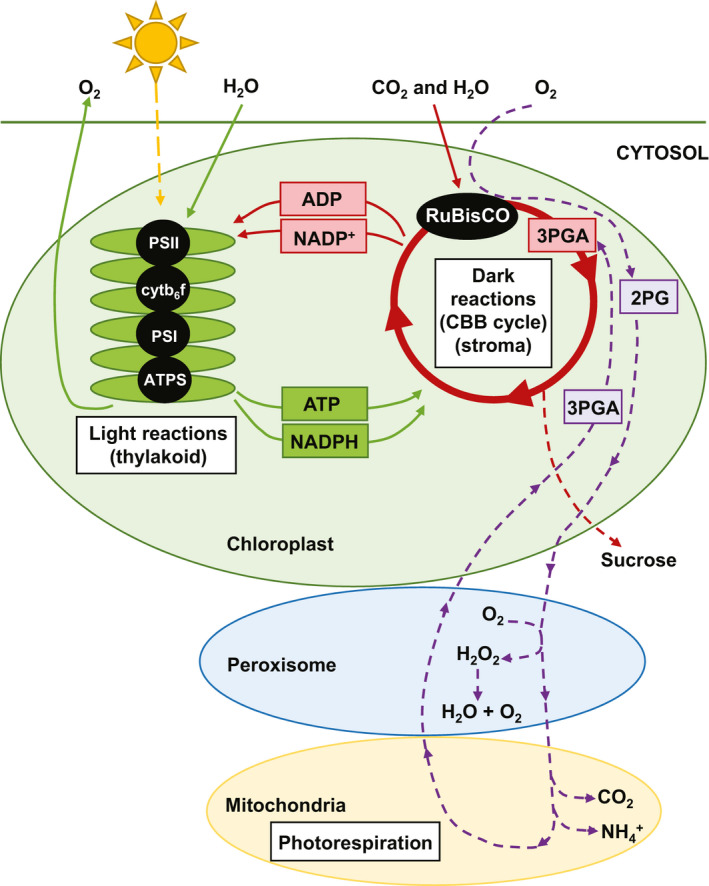
Generalized schematic diagram of photosynthetic light and dark reactions, as well as photorespiration, in C_3_ plants. Light reactions are depicted in green, dark reactions are indicated in red, and the photorespiratory pathway is shown in purple. Major enzymes are indicated in black ovals. 2PG, 2‐phosphoglycolate; 3PGA, 3‐phosphoglycerate; ADP, adenosine diphosphate; ATP, adenosine triphosphate; ATPS, ATP synthase; CBB, Calvin–Benson–Bassham; cytb_6_f, cytochrome b_6_f complex; NADP(H), nicotinamide adenine dinucleotide phosphate; PSI, photosystem I; and PSII, photosystem II

Crop yield in relation to photosynthesis is governed by exposure to sunlight throughout a growing season and the efficiency with which the plant can capture this light energy, use it to convert CO_2_ into carbohydrates, translate the resulting energy into biomass, and for grain/seed/fruit crops, partition this energy into harvested fractions (Jansson et al., 2018). Photosynthesis in C_3_ plants, which includes all legumes, comprises two sets of reactions, termed the light and dark reactions (Figure 1). The light reactions take place in the thylakoid membrane system and involve the capture of solar energy by chlorophyll and other associated pigments, the splitting of water, and electron transport, which leads to the production of NADPH and ATP. The dark reactions then use this NADPH and ATP to fuel the Calvin cycle, which allows CO_2_ to be assimilated into carbohydrate (Long, Marshall‐Colon, & Zhu, 2015). During C_3_ photosynthesis, CO_2_ fixation via the Calvin cycle begins with the carboxylation of ribulose 1,5‐bisphosphate (RuBP) through the activity of ribulose 1,5‐bisphosphate carboxylase–oxygenase (RuBisCO), leading to the production of two 3‐phosphoglycerate (3PGA) molecules. Due to its exceptionally slow catalytic rate (Parry et al., 2013), up to 50% of total soluble leaf protein is typically invested in RuBisCO to compensate for its inadequate kinetics (Erb & Zarzycki, 2018), rendering this process highly inefficient in terms of nitrogen usage. Furthermore, at current atmospheric levels, O_2_ competes with CO_2_ at the active site of RuBisCO, leading to the oxygenation of RuBP and the subsequent photorespiratory pathway that recycles the resulting oxygenation products (Figure 1). This pathway results in a substantial penalty on net photosynthetic efficiency, causing losses of up to 30% in terms of net carbon assimilation in C_3_ plants (Zhu, Long, & Ort, 2010), with even more pronounced costs at elevated temperatures (Long, Marshall‐Colon, & Zhu, 2015).

The efficiency of carboxylation and the net losses incurred through photorespiration depend upon the relative partial pressures of CO_2_ and O_2_ at the active site of RuBisCO (Lorimer, 1981). Since C_3_ plants are not photosynthetically saturated at current [CO_2_], and likely will not be until the middle to end of this century (Tausz et al., 2013), it is expected that during this timeframe, photosynthetic rates would increase under e[CO_2_], while photorespiratory losses would be reduced (Drake, Gonzalez‐Meler, & Long, 1997; Kimball, 2016). In most cases, in the absence of other limiting factors and under saturating light, this is indeed the case in the short term (up to approximately 2 weeks), whereby e[CO_2_] leads to increased photosynthetic efficiency compared to ambient [CO_2_]. However, over the long term, this initial stimulatory response to e[CO_2_] is typically not maintained, and photosynthetic capacity eventually declines, at least to some extent, in a process termed “down‐regulation” or “acclimation” (e.g., Ainsworth & Rogers, 2007; Aranjuelo et al., 2005; Reich et al., 2018; Rogers & Ellsworth, 2002; Sage, Sharkey, & Seemann, 1989). While modest increases in photosynthetic rates and biomass production are often still observed under long‐term CO_2_ enrichment, these gains are much less substantial than they would be in the absence of photosynthetic acclimation. Harnessing the potential of legumes to maximize photosynthetic and associated biomass gains under e[CO_2_] could therefore provide a possible means for generating high‐yielding crops in the future.

While reductions in stomatal conductance, which is a key determinant of the concentration of CO_2_ at RuBisCO active sites in C_3_ plants, are typically observed under long‐term e[CO_2_] (Ainsworth & Rogers, 2007), this response does not appear to limit intercellular [CO_2_] and is therefore not believed to contribute to photosynthetic acclimation (Ainsworth & Long, 2005; Aranjuelo et al., 2005). Instead, it has been suggested that non‐diffusional factors related to alterations in RuBisCO levels, activity, and/or degradation are more likely to be responsible for this phenomenon (Irigoyen et al., 2014). Although the precise mechanisms driving photosynthetic acclimation under e[CO_2_] have yet to be elucidated in full, two main models have been proposed, including the “sink limitation” and “N limitation” theories, which are not mutually exclusive (Tausz et al., 2013).

Sink tissues are net importers of photosynthetic products from source leaves and typically comprise all belowground organs of a plant, along with certain aboveground organs such as developing shoots and seeds (Ludewig & Sonnewald, 2016). In the case of sink limitation, initial increases in the rate of carbon assimilation stemming from e[CO_2_] are believed to eventually lead to an accumulation of non‐structural carbohydrates in leaf tissues as a result of replete sinks and a lack of additional sink capacity (i.e., the ability to generate new sinks and/or expand existing sinks; Erice et al., 2011; Lewis et al., 2002). This build‐up of carbohydrates to saturating levels may be responsible for the transcriptional down‐regulation of several genes encoding enzymes involved in photosynthesis, such as RuBisCO (Moore et al., 1999; Sheen, 1994), resulting in a negative feedback effect on the photosynthetic process. Correspondingly, photosynthetic acclimation has been found to be more pronounced when plants are grown in small pots where root growth, and hence sink activity, is limited (Ainsworth et al., 2002).

The nitrogen limitation theory, on the other hand, derives from the fact that acclimation to e[CO_2_] also tends to be associated with diminished leaf nitrogen concentrations (Leakey et al., 2009). Since nitrogen uptake is not augmented during initial biomass expansions that are typically observed under e[CO_2_], and may even decrease following long‐term exposure to e[CO_2_] (Guo, Sun, Li, Liu, et al., 2013; Parvin et al., 2018), the amount of existing nitrogen must be distributed over a more substantial quantity of tissue. This results in a dilution of its concentration and leads to a nutrient deficiency that hinders plant growth (Tausz et al., 2013). Decreased nitrogen partitioning to leaves (Seneweera, 2011) and a smaller proportion of the available nitrogen being allocated to RuBisCO synthesis under e[CO_2_] (Drake, Gonzalez‐Meler & Long, 1997; Sage, Sharkey, & Seemann, 1989) likely also contribute to reductions in leaf nitrogen concentrations. While declines in RuBisCO and other photosynthetic enzymes, as well as reduced leaf nitrogen concentrations, are clearly associated with photosynthetic acclimation under e[CO_2_], the specific mechanisms behind these phenomena remain to be resolved and it is unclear whether they are a direct cause, or simply a consequence, of this process (Gamage et al., 2018).

**Figure 2:**
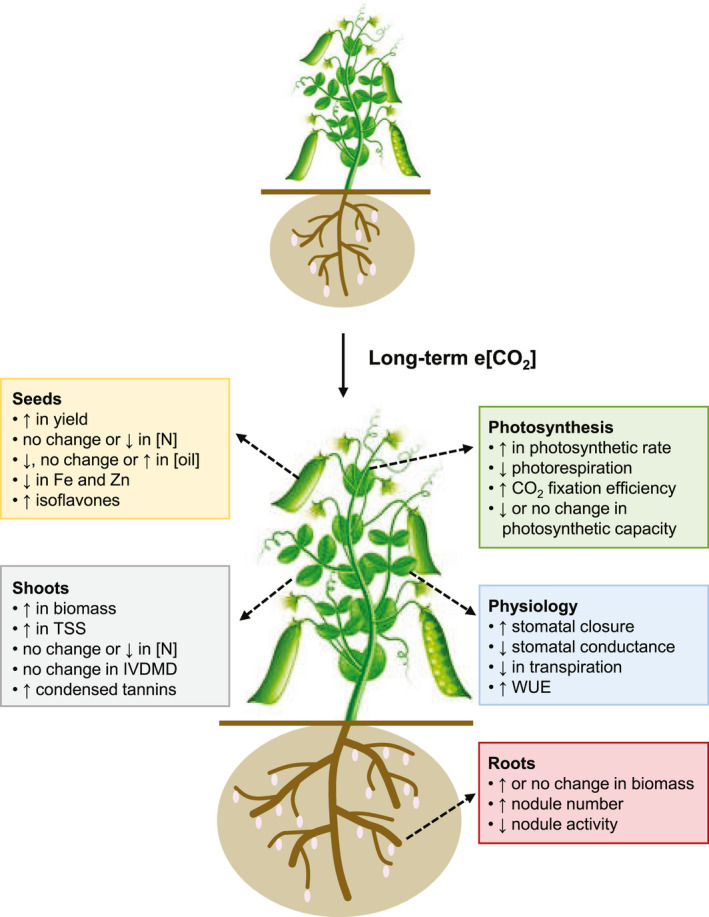
Typical growth and quality effects of long‐term e[CO_2_] exposure in legume species grown under field conditions. Fe, iron; IVDMD, in vitro dry matter digestibility; N, nitrogen; TSS, total soluble sugars; WUE, water‐use efficiency; and Zn, zinc

Due to their unique physiology, legumes often appear to be especially responsive to long‐term e[CO_2_] exposure in the form of increased photosynthetic rates and reduced susceptibility to photosynthetic acclimation, as well as associated gains in vegetative biomass, compared to non‐nodulating plants (e.g., Ainsworth et al., 2002; Ainsworth et al., 2004; Jablonski, Wang, & Curtis, 2002; Lee, Barrott, & Reich, 2011; Lee et al., 2003; Scheelbeek et al., 2018; Figure 2, Tables 1 and 2; Table S1). This may stem, at least in part, from the presence of root nodules and their associated symbiotic relationship with N_2_‐fixing bacteria, which provides an additional strong sink for carbohydrates and allows plants to be less constrained by soil nitrogen limitations, thereby ameliorating their capacity to maintain foliar nitrogen concentrations under e[CO_2_] (Cabrerizo et al., 2001; Lam et al., 2012; Rogers et al., 2006). Correspondingly, aboveground biomass has been found to be enhanced under long‐term e[CO_2_] in nodulated *Medicago truncatula*, but not an N_2_‐fixation‐deficient mutant (Guo, Sun, Li, Liu, et al., 2013).

**Table 1:** Examples of effects of long‐term^a^ e[CO_2_] on photosynthetic rate and biomass production in a selection of forage and other non‐grain legumes

Legume	No.	Photosynthetic rate (Agrowth µmol m^−2^ s^−1^)	Biomass
Forage legumes			
Alfalfa	2^GH^	79% ↑ in A_growth_ in non‐mycorrhizal plants 62% ↑ in A_growth_ in mycorrhizal plants	37% ↑ in shoot DW in non‐mycorrhizal plants 31% ↑ in shoot DW in mycorrhizal plants
	3^GC^	50% ↑ or no change in A_growth_ at 56 d depending on rhizobial strain at 56 d	↑ in shoot DW at 56 d with both rhizobial strains ↑ or no change in root DW at 56 d depending on rhizobial strain
	4^GH^	ND	no change in shoot DM
	6^GH^	↑ in A_growth_ acclimation in mycorrhizal, but not non‐mycorrhizal, plants	38% ↑ in leaf DM in non‐mycorrhizal plants, no change in mycorrhizal plants 55% ↑ in root DM in mycorrhizal plants, no change in non‐mycorrhizal plants
	8^b^ ^,GC^	no change in A_growth_ at 25°C	84% ↑ in leaf DW at 25°C 108% ↑ in stem DW at 25°C 84% ↑ in root DW at 25°C
	9^b^ ^,GC^	39% ↑ in A_growth_ with addition of 15 mM NH_4_NO_3_, no change with 0 mM or 10 mM NH_4_NO_3_ acclimation with 0 mM and 15 mM NH_4_NO_3_, no acclimation with 15 mM NH_4_NO_3_	no change in total DW with 0 mM NH_4_NO_3_, 44% and 39% ↑ total DW with 10 mM and 15 mM NH_4_NO_3,_ respectively
White clover (*Trifolium repens*)	10^b^ ^,GC^	ND	73% and 49% ↑ in leaf DW at 36 d without and with exogenous N, respectively 26% and 19% ↑ in root DW at 36 d without and with exogenous N, respectively
	11^b^ ^,GH^	no change in A_growth_	75% ↑ in canopy DW
	12^b^ ^,F^	ND	11%–20% ↑ in biomass yield regardless of cutting frequency or level of N (low or high)
	13^OP^	ND	40% ↑ in ABG DW
Subclover (*Trifolium subterraneum*)	14^TF^	12% ↑ in A_growth_	19% ↑ in total shoot biomass ↑ in root biomass
Berseem (*Trifolium alexandrinum*)	16^OP^	92%–107% ↑ in A_growth_ depending on growth stage	40%–98% ↑ in total shoot FW depending on growth stage
Sainfoin (*Onobrychis viciifolia*)	17^GC^	ND	25% ↑ in total DW 36% ↑ in leaf DW 25% ↑ in root DW
Birdsfoot trefoil (*Lotus corniculatus*)	18^b^ ^,GC^	ND	↑ in shoot DW 82% ↑ in root DW
Kidney vetch (*Anthyllis vulneraria*)	19^b^ ^,GC^	ND	no change in shoot or root DW
Other non‐grain legumes			
Acacia (*Acacia melanoxylon*)	50^b^ ^,GCH^	↑ in A_growth_ only at two highest N levels no acclimation observed regardless of N level	no change in total DW at low N, ~100% ↑ at moderate and high N
*Medicago truncatula*	52^OP^	ND	37% ↑ in ABG DW in nodulated plants no change in ABG DW in non‐nodulating mutants
*Medicago minima*	53^b^ ^,GH^	no change in A_growth_	no change in leaf or root DW
*Medicago glomerata*	54^b^ ^,GH^	no change in A_growth_	no substantial change in leaf or root DW
Wild lupine (*Lupinus perennis*)	55^b^ ^,GC^	39% average ↑ in A_growth_ regardless of N level acclimation observed regardless of N level	80% average ↑ in total DW regardless of N level

Note

Results are from studies carried out at ambient temperatures under non‐limiting conditions unless otherwise noted.

Abbreviations: ABG, aboveground; A_growth_, net photosynthetic rate at saturating (or near‐saturating) light under growth [CO_2_]; d, days; DW, dry weight; ^F,^ free‐air CO_2_ enrichment; FW, fresh weight; ^GC,^ growth cabinet; ^GCH,^ growth cabinet hydroponic; ^GH,^ greenhouse; N, nitrogen; ND, no data; No., number assigned to study in Supplemental Table 1; ^OP,^ open‐top chamber in pots; ^TF,^ temperature gradient enclosure sown in field.

^a^ Treated for >3 weeks.

^b^ At least a subset of plants received exogenous N throughout experiment.

**Table 2:** Examples of effects of long‐term^a^ e[CO_2_] on photosynthetic rate, biomass production, and seed yield in a selection of grain and groundnut legumes

Legume	No.	Photosynthetic rate	Biomass	Seed yield
Lenti (*Lens culinaris*) l	20^F^	ND	•18% average ↑ in ABG biomass across cultivars at flowering 50% average ↑ in ABG biomass across cultivars between flowering and maturity	18%–138% average ↑ across cultivars depending on year
	21^F^	ND	ND	59% ↑
	22^F^	29%–55% ↑ in A_growth_ depending on cultivar	31%–34% ↑ in ABG at maturity depending on cultivar no change in root biomass at maturity	55% ↑ across cultivars
Faba bean (*Vicia faba*)	23^F^	ND	ND	59% ↑
	24^CF^	46% ↑ in canopy photosynthetic rate	58% ↑ in ABG biomass	51% ↑
Soybean	26^F^	17%–26% average ↑ in A_growth_ across cultivars depending on growth stage	13%–32% average ↑ in ABG biomass across cultivars depending on year	0%–20% average ↑ across cultivars depending on year
	27^OP^	ND	4%–53% ↑ in ABG biomass at maturity depending on cultivar	3%–95% ↑ depending on cultivar
	28^F^	ND	15%–17% ↑ in ABG net primary production depending on year	15%–16% ↑ across years
	29^b^ ^,TP^	14% average ↑ in A_growth_ across cultivars at flowering stage	7%–72% ↑ in ABG biomass at maturity depending on cultivar and year	0%–62% ↑ depending on cultivar
	30^OF^	ND	up to 61% ↑ in stem DW at maturity	no change
	31^F^	↑ in A_growth_ acclimation observed	no change	no change
	33^b^ ^,OP^	ND	22%–87% ↑ in shoot DW depending on cultivar 19% average ↑ in root length across cultivars	12%–91% ↑ depending on cultivar
	34^b^ ^,OP^	27%–46% ↑ in A_growth_ depending on growth stage and year	15% average ↑ in ABG biomass across years	13% average ↑ across years
Mung bean (*Vigna radiata*)	37^F^	7%–19% ↑ in A_growth_ depending on growth stage	11% ↑ in total biomass	14% ↑
	38^OF^	ND	ND	59% ↑
Field pea (*Pisum sativum*)	39^F^	ND	19%–33% ↑ in DW biomass across cultivars depending on growth stage	26% average ↑ across cultivars
Chickpea (*Cicer arietinum*)	41^OF^	ND	↑ in stem and leaf DW	10%–13% ↑ depending on year
	43^OF^	ND	48%–142% ↑ in stem DW at 50% flowering depending on year 60%–260% ↑ in leaf DW depending on year	ND
	44^OP^	ND	33% average ↑ in leaf DW across growth stages 37% average ↑ in shoot DW across growth stages	no change
French bean (*Phaseolus vulgaris*)	45^OF^	ND	22%–41% ↑ in total ABG DW depending on cultivar at 50% flowering 35%–54% ↑ in total ABG DW depending on cultivar at peak fruiting stage 85%–150% ↑ in root DW depending on cultivar and growth stage	↑ in both cultivars
Kidney bean (*Phaseolus vulgaris*)	46^b^ ^,GH^	↑ in A_growth_ at all growth stages acclimation observed during reproductive growth	27% ↑ in total vegetative DW at maturity	34% ↑
Pigeon pea (*Cajanus cajan*)	48^OF^	30%–41% ↑ in A_growth_ depending on growth stage and year no acclimation	29% average ↑ in total DW across years at harvest	29% average ↑ across years
	49^OF^	14%–29% ↑ in A_growth_ depending on cultivar	11% ↓ to 109% ↑ in total vegetative DW depending on cultivar no change in root DW	43%–185% ↑ depending on cultivar
Peanut (*Arachis hypogaea*)	51^GHF^	up to 40% ↑ in A_growth_	16% average ↑ in ABG DW across years No change in root DW	25% average ↑ across years

Note

Results are from studies carried out at ambient temperatures without intentional drought treatments.

Abbreviations: ABG, aboveground; A_growth_, net photosynthetic rate at saturating (or near‐saturating) light under growth [CO_2_], ^CF,^ crop enclosure in field; DW, dry weight; ^F,^ free‐air CO_2_ enrichment; ^GC,^ growth cabinet; ^GH,^ greenhouse; ^GHF,^ greenhouse sown in field; N, nitrogen; ND, no data; No., number assigned to study in Table S1; ^OF,^ open‐top chamber sown in field; ^OP,^ open‐top chamber in pots; and ^TP,^ temperature gradient enclosure sown in pots.

^a^ Treated for >3 weeks.

^b^ At least a subset of plants received exogenous N throughout experiment.

However, results have been conflicting (Table 1), and at least under certain circumstances, nodulated leguminous species have been found to undergo acclimation in a manner similar to non‐N_2_‐fixing plant species (e.g., Aranjuelo et al., 2005; Lee, Barrott, & Reich, 2011; Sanz‐Sáez et al., 2010; Xu, Gifford, & Chow, 1994). Various limiting factors, including inadequate water supply (Aranjuelo et al. 2009), low intensity of solar radiation (Ainsworth et al., 2003), and insufficient phosphorus availability (Duchein, Monicel, & Betsche, 1993), as well as other environmental pressures, may contribute to such findings. However, in at least a proportion of cases, it appears that the presence of nodules alone cannot support the high sink and/or nitrogen demands required under e[CO_2_]. Indeed, although some studies have found foliar nitrogen concentrations to be maintained or increased under e[CO_2_] in legumes (e.g., Sanz‐Sáez et al., 2010; Parvin et al., 2018), reductions in foliar nitrogen levels are just as commonly encountered (e.g., Bertrand et al., 2007; Bourgault et al., 2016; Bourgault et al., 2017) albeit often to a lesser extent than their non‐leguminous C_3_ counterparts (e.g., Lam et al., 2012). While there is evidence that unlike non‐N_2_‐fixing plants, net photosynthetic rates may not be tightly linked to foliar nitrogen concentrations in plants that fix N_2_ (Adams et al., 2016), supplementation with exogenous nitrogen fertilizer has been found to alleviate photosynthetic acclimation in some legumes (Sanz‐Sáez et al., 2010; Schortemeyer et al., 1999). This supports the notion that at least under certain circumstances, N_2_‐fixation is inadequate to maintain growth rates under long‐term e[CO_2_].

In line with this, photosynthetic acclimation and growth responses under e[CO_2_] in various legume species have been found to correlate with their capacity for nodule formation (Ainsworth et al., 2004; Cernusak et al., 2011; Guo, Sun, Li, Liu, et al., 2013). As such, variations in this trait could be responsible for at least a proportion of the genotypic and/or interspecific differences observed under CO_2_ enrichment (West et al., 2005). Although relatively little is currently known concerning the role of nodulation in photosynthetic acclimation to e[CO_2_], it appears that nodule numbers and mass typically increase under these conditions (e.g., Cabrerizo et al., 2001; Lam et al., 2012; Lee et al., 2003; Sreeharsha, Sekhar, & Reddy, 2015). Despite these increases, however, it has been suggested that feedback inhibition of rhizobial nitrogenase activity under e[CO_2_] may lead to an overall down‐regulation of nodule activity and thus N_2_‐fixation (Hartwig et al., 1994), which could also contribute to the photosynthetic acclimation sometimes seen in legumes grown under CO_2_ enrichment.

The strain of rhizobial inoculant can also have a substantial impact on a legume’s response to e[CO_2_] (Bertrand et al., 2007; Sanz‐Sáez, Erice, Aguirreolea, Muñoz, et al., 2012), possibly through an increased efficiency of N_2_‐fixation in particular symbiont/legume genotype combinations (Provorov & Tikhonovich, 2003). However, this observation has not always held true, and the use of an enhanced N_2_‐fixing inoculant with soybean in Free‐Air CO_2_ Enrichment (FACE) field experiments, for example, did not enhance photosynthesis, growth or seed yield under e[CO_2_] or ambient [CO_2_] compared to a typical inoculant (Sanz‐Sáez et al., 2015). This lack of stimulation was suggested to result, at least in part, from competition with native rhizobia in the soil, which could be one cause of discrepancies observed between experiments carried out in pots (e.g., Bertrand et al., 2007) versus field conditions. However, it is also possible that a reduced investment in nodule biomass resulting from the use of a rhizobial strain exhibiting a particularly high N_2_‐fixation efficiency may lead to a reduction in nodule sink strength, thus limiting the plant’s ability to take advantage of e[CO_2_] conditions (Sanz‐Sáez et al., 2015).

In addition to their relationship with rhizobia, legumes can also establish symbioses with arbuscular mycorrhizal fungi, which promote nutrient uptake (particularly phosphorus) and act as an additional carbon sink (Mortimer, Pérez‐Fernández, & Valentine, 2008). This implies that their presence might also prevent photosynthetic acclimation and enhance biomass production under e[CO_2_] in leguminous species. However, results have been highly variable to date (e.g., Baslam, Erice, & Goicoechea, 2012; Gavito et al., 2000; Goicoechea et al., 2014; Jakobsen et al., 2016; Olesniewicz & Thomas, 1999), which may derive from differences in experimental conditions, including genotype/species assessed, level of CO_2_ to which the plants were subjected, and rooting volume.

The importance of the source/sink ratio on the propensity for photosynthetic acclimation under e[CO_2_] in legumes has also been demonstrated by the fact that the cutting or grazing of a legume crop, which is the typical manner in which forage legumes such as alfalfa are managed, can improve photosynthetic response (Erice et al., 2006a) and enhance biomass gains under long‐term e[CO_2_] (Erice et al., 2006b). This occurs because the removal of shoots, which normally act as source organs that provide carbohydrates to sink tissues such as roots and young developing shoots, initiates the mobilization of carbon and nitrogen reserves from roots to shoots (Avice et al., 2003). This essentially results in an inversion of source and sink organs, leading to the generation of a new strong sink. Defoliation also triggers the degeneration of existing nodules in legumes (Vance et al., 1979), and their regeneration gives rise to yet another sink (Erice et al., 2006a).

## 3 EFFECT OF ELEVATED [CO_2_] ON LEGUME SEED YIELD

Although increases in net photosynthetic rates and biomass production are often observed under long‐term e[CO_2_], such responses have not always been found to correlate well with reproductive traits across plant species in general (Ackerly & Bazzaz, 1995; Farnsworth & Bassaz, 1995). Therefore, seed yield responses under e[CO_2_] are not necessarily easy to predict. In the majority of legumes assessed thus far, seed yields tend to increase under long‐term e[CO_2_] compared to ambient [CO_2_] (Figure 2, Table 2), mainly as a result of greater pod numbers rather than the number of seeds per pod or individual seed weight (Ainsworth et al., 2002; Bourgault et al., 2016; Kumagai et al., 2015; Li et al., 2019). However, in many instances, these enhancements are often less substantial than those observed in vegetative biomass, and even less so than increases in photosynthesis (e.g., Ainsworth et al., 2002; Bishop et al., 2015; Dijkstra, Schapendonk, & Groenwold, 1993; Prasad et al., 2002). These findings likely arise due to the reductions in harvest index (proportion of total biomass that is partitioned into the seeds) that are frequently observed under CO_2_ enrichment (Ainsworth et al., 2002; Bishop et al., 2015; Kumagai et al., 2015; Morgan et al., 2005).

To further complicate matters, environmental factors appear to play a substantial role in seed yield effects under e[CO_2_], which is evidenced by the finding that smaller stimulations in yield are typically observed in FACE experiments compared to those conducted in controlled environments (Ainsworth et al., 2008). Furthermore, a large amount of intraspecific variability is evident in legume species with respect to the magnitude of yield gains under e[CO_2_] (Bishop et al., 2015; Bunce, 2016; Kumagai et al., 2015; Li et al., 2017; Li et al., 2019; Vanaja et al., 2015). Intriguingly, it has been found that soybean cultivars displaying the greatest yield increases under CO_2_ enrichment were not necessarily those with the highest yields under ambient [CO_2_], but instead those that displayed the greatest yield gain under e[CO_2_] (Li et al., 2017; Li et al., 2019). These genotypic disparities have been attributed to several potential characteristics, with those genotypes exhibiting the greatest seed yield responses under e[CO_2_] often possessing larger seeds (Sicher, Bunce, & Matthews, 2010), higher harvest index (or ability to maintain harvest index at e[CO_2_]; Bishop et al., 2015), or capacity to increase main stem node numbers, and hence sites for pod production, under e[CO_2_] (Bunce, 2016) compared to cultivars that performed less favorably under such conditions. It has also been suggested that indeterminate genotypes may provide superior seed yield responses under e[CO_2_] compared to their determinate counterparts (Kumagai et al., 2015). However, this did not appear to be a dominant factor when comparisons were made across a broad range of soybean cultivars (Kumagai et al., 2015) and differences in the magnitude of seed yield stimulation under e[CO_2_] are also observed among cultivars displaying the same growth habit (e.g., Lam et al., 2012).

As is the case for photosynthetic and biomass amplification under e[CO_2_], seed yield gains also appear to be closely related to plant nitrogen concentrations. Indeed, it has been found that the content of fixed nitrogen in soybean seeds, and hence symbiotic N_2_‐fixation during the reproductive period, is tightly linked to seed yield increases under e[CO_2_] (Li et al., 2019). However, despite the ability of legumes to fix nitrogen, and their overall superior improvements in photosynthesis and biomass accumulation under e[CO_2_] compared to non‐leguminous species (Ainsworth et al., 2002; Ainsworth et al., 2004), the stimulation of soybean seed yield does not differ substantially from that of other non‐N_2_‐fixing C_3_ crops such as wheat (*Triticum aestivum*) and rice (*Oryza sativa*) (Bishop, Leakey, & Ainsworth, 2014). This supports the notion that N limitation may still contribute to the inability of some legumes to maximize yield responses under e[CO_2_], and suggests that disparities in N_2_‐fixation capacity, or the ability to maintain nitrogen levels specifically in seeds, may also contribute to cultivar‐ and species‐specific differences in yield gains under these conditions.

Taken together, these findings indicate that legume seed yield responses to e[CO_2_] may be influenced by a variety of factors, including sink strength, N_2_‐fixation efficiency, and the translocation of resources to the seeds. In soybean at least, it has been suggested that historically, breeders may have unintentionally selected for genotypes that are less stimulated by e[CO_2_] in terms of seed yields. This is evidenced by the fact that at least certain older genotypes have been found to be more responsive to e[CO_2_] than modern cultivars (Leakey & Lau, 2012; Ziska, Bunce, & Caulfield, 2001). Although the specific traits that best predict maximum yield responses to e[CO_2_] in legume species (or other crop species) remain unclear (Bishop et al., 2015; Leakey & Lau, 2012; Ziska et al., 2012), the failure of seed yield increases to parallel the potential provided by the stimulation of canopy photosynthetic rates under e[CO_2_] implies that there is substantial potential to improve the capacity of legumes to better take advantage of future atmospheric conditions.

## 4 EFFECT OF ELEVATED [CO_2_] ON LEGUME QUALITY

Although the effect of e[CO_2_] on photosynthesis and associated yield traits in plants has been by far the most well studied thus far, elevated levels of CO_2_ have also been found to lead to other consequences that could have serious deleterious outcomes in terms of crop quality. These include reductions in protein concentrations (Fernando et al., 2015), as well as declines in the levels of certain vitamins and other macro‐ and micronutrients (Högy & Fangmeier, 2008; Myers et al., 2014; Figure 2). While all of these effects would be disadvantageous to legume production overall, whether a crop is harvested as a forage (high protein vegetative tissue), oilseed (high oil seed) or pulse (high protein seed), will determine the precise components that are of greatest concern in each case.

### 4.1 Forage quality

In the case of leguminous forage species, two important parameters for shoot quality include the concentrations of non‐structural carbohydrates, which provide a source of rapidly fermentable energy, and crude protein, which supplies the nitrogen building blocks necessary for the production of other proteins by microbial populations within the rumen from which livestock‐derived products such as meat and dairy are derived. As discussed previously, the long‐term exposure of legumes to e[CO_2_] can lead to a reduction in nitrogen concentrations in leaf tissues, at least when they are grown under limiting conditions (Aranjuelo et al., 2005; Sanz‐Sáez et al., 2010; Table 3). Unsurprisingly, reductions in leaf nitrogen concentrations are typically associated with a decline in crude protein levels, which has the potential to decrease forage quality and digestibility (Milchunas et al., 2005). Ruminants require at least 70 g protein/kg dry matter (DM) for maintenance, 100–140 g protein/kg DM for growth, and 150 g protein/kg DM for lactation (Izaurralde et al., 2011). Since leguminous forages tend to possess relatively high crude protein values ranging between approximately 170 and 310 g protein/kg DM (Fulkerson et al., 2007; Lee, 2018), their quality may be less impacted by small drops in crude protein than their non‐leguminous counterparts. However, if crude protein levels declined to a great enough extent, feed derived from even leguminous forages would need to be supplemented with additional nitrogen to offset these losses under future atmospheric conditions, which would increase the cost of production substantially (Izaurralde et al., 2011).

**Table 3:** Examples of effects of long‐term^a^ e[CO_2_] on the quality of forage legumes and their close relatives

Legume	No.	Foliar [N]	Foliar [soluble protein] or [crude protein]	Foliar [TSS] or [NSC]	Lignin	ADF/NDF	In vitro digestibility	Other
Alfalfa	1^GH^	ND	↓ in both mycorrhizal and non‐mycorrhizal plants	no change	no change in non‐mycorrhizal plants ↓ in mycorrhizal plants	ND	ND	↑ in methane emissions
	3^GC^	↓ with both rhizobial strains	ND	ND	ND	↑ or ↓ depending on rhizobial strain	no change with either rhizobial strain	ND
	4^GH^	ND	↓ or no change depending on rhizobial strain	no change with either rhizobial strain	↑ or no change depending on rhizobial strain	↑ or no change depending on rhizobial strain	no change with either rhizobial strain	ND
	6^GH^	↓ in mycorrhizal and non‐mycorrhizal plants	↑ in mycorrhizal plants ↓ in non‐mycorrhizal plants	↑ in mycorrhizal plants ↓ in non‐mycorrhizal plants	ND	ND	ND	ND
	8^b^ ^,GC^	ND	no change at 25°C	ND	ND	ND	ND	ND
	9^b^ ^,GC^	↑ at all N levels; greatest ↑ at highest NH_4_NO_3_ level	no change at any N level	↑ with 0 and 10 mM NH_4_NO_3_ no change with 15 mM NH_4_NO_3_	ND	ND	ND	ND
White clover	10^b^ ^,GC^	11% and 21% ↓ at 36 d without and with exogenous N, respectively	ND	ND	ND	ND	ND	ND
	13^OP^	↓ early in regrowth no change later in regrowth	ND	ND	ND	ND	ND	↓ in K and S
Subclover	15^TF^	10% ↓	ND	28% average ↑	ND	ND	no consistent change across years	ND
Berseem	16^OF^	up to 33% ↓	up to 26% ↓	up to 33% ↑ in organic [C]	ND	ND	ND	↑ in P
Sainfoin	17^GC^	34% ↓	ND	ND	ND	ND	ND	ND
*Medicago truncatula*	52^OP^	↑ in nodulating plants ↓ in non‐nodulating mutants	ND	↑ in nodulating plants and non‐nodulating mutants	ND	ND	ND	ND
*Medicago minima*	53^b^ ^,GH^	no change	ND	no change	ND	ND	ND	ND
*Medicago glomerata*	54^b^ ^,GH^	no change	ND	no change	ND	ND	ND	ND

Note

Results are from studies carried out at ambient temperatures under non‐limiting conditions unless otherwise noted.

Abbreviations: ADF, acid detergent fiber; d, days; ^GC,^ growth cabinet; ^GH,^ greenhouse; K, potassium; N, nitrogen; ND, no data; NDF, neutral detergent fiber; No., number assigned to study in Table S1; NSC, non‐structural carbohydrates; ^OF,^ open‐top chamber sown in field; ^OP,^ open‐top chamber in pots; ^TF,^ temperature gradient enclosure sown in field; P, phosphorus; S, sulfur; and TSS, total soluble sugars.

^a^ Treated for >3 weeks.

^b^ At least a subset of plants received additional N as fertilizer throughout experiment.

While high levels of crude protein are beneficial in terms of forage quality, proteins from leguminous forages tend to be digested at a much higher rate within the rumen than the plant cell wall components (cellulose and hemicellulose) that make up the main source of a forage’s energy. This asynchrony leads to energy limitation for the ruminal microbial population due to a surplus of forage proteolysis products and a shortage in cell wall breakdown products (reviewed by Singer, Weselake, & Acharya, 2018). A substantial proportion of this superfluous nitrogen is then excreted onto pasture land in urine, resulting in nitrogen losses of up to 70% (Kingston‐Smith, Marshall, & Moorby, 2013). As such, it has been suggested that increased concentrations of rapidly digestible soluble carbohydrates in forages could theoretically improve quality by augmenting the availability of a rapidly digestible energy source to balance forage proteolysis and fuel livestock protein biosynthesis (Brito et al., 2008). Under e[CO_2_] conditions, elevations in non‐structural carbohydrates and C:N ratios are typically seen in foliage (e.g., Aranjuelo et al., 2005; Sanz‐Sáez et al., 2010), which could improve quality and lead to increased nitrogen use efficiency (Bertrand et al., 2007). Nitrogen excreted by livestock can also lead to the leaching of nitrates into groundwater and/or act as substrate for the generation of the potent greenhouse gas, N_2_O, which are both environmentally deleterious (Oenema et al., 1997; Wachendorf et al., 2008). Therefore, the boost in readily digestible carbohydrates typically observed under e[CO_2_] could also serve to minimize such negative environmental impacts of livestock production.

The presence of relatively low concentrations (between 3% and 4% DM) of polyphenolic condensed tannins in the vegetative tissues of forages is also highly beneficial to ruminant production due to their ability to complex with plant proteins, thus slowing down their degradation within the rumen and improving nitrogen use efficiency (Aerts, Barry, & McNabb, 1999). As is the case with increased non‐soluble carbohydrate concentrations, this leads to enhancements in the production of meat and milk products (McMahon et al., 2000), as well as reductions in greenhouse gas emissions resulting from ammonium excreted in urine (Smith et al., 2008). In addition, their presence has also been found to reduce the incidence of pasture bloat (Tanner et al., 1995), and condensed tannin‐containing legumes such as sainfoin, sulla (*Hedysarium coronarium*), and birdsfoot trefoil are therefore gaining popularity as forages (Acharya et al., 2013). Since condensed tannins are carbon‐based and do not contain nitrogen, it is not surprising that their concentration has been found to increase under e[CO_2_] due to increased carbon availability (Carter, Theodorou, & Morris, 1999; Stiling & Cornelissen, 2007). Unfortunately, at levels above an approximately 5% DM threshold, condensed tannins become anti‐nutritional due to associated reductions in voluntary intake (McSweeney et al., 2001) and impairment of rumen function (Kumar & Singh, 1984). Concentrations rarely reach these levels in condensed tannin‐containing leguminous forages under current environmental conditions; however, growth under e[CO_2_] has been found to increase condensed tannin levels on average 22% in plants (Robinson, Ryan, & Newman, 2012). Therefore, it is possible that future atmospheric conditions could tip quantities over the limit, and further research in this area, along with careful monitoring in the future, is warranted.

Inefficiencies in the fermentation of forages within the rumen also contribute to the livestock‐derived emission of methane, which is another greenhouse gas that drives climate change (IPCC, 2007). This occurs through the digestion of cellulosic forage components by microorganisms within the rumen, which produce hydrogen as a by‐product that inhibits further fermentation. As a means of preventing reductions in fermentation, the hydrogen (along with CO_2_) is converted to methane by rumen methanogens. This process utilizes between 2% and 15% of the energy consumed by a ruminant, and the methane is then excreted into the atmosphere (Van Nevel & Demeyer, 1996). Although very little is currently known regarding the effects of e[CO_2_] on this process, there is some evidence that CO_2_ enrichment may lead to increases in the potentiality of a forage for eliciting methane emissions (Baslam et al., 2013), which would be very detrimental in the context of climate change.

Fiber concentration, which is often estimated as neutral detergent fiber (NDF; comprising cellulose, hemicellulose, and lignin), acid detergent fiber (ADF; comprising cellulose and lignin), and acid detergent lignin (ADL), can also impact forage digestibility. Lignin in particular is known to be largely indigestible to ruminants, and decreases in this compound are therefore associated with improved forage quality (Irigoyen et al., 2014). Unfortunately, results of studies aimed at assessing the effect of e[CO_2_] on forage cell wall components have been highly variable and appear to be heavily dependent upon the rhizobial strain used and the presence of mycorrhizal fungi (Baslam et al., 2013; Bertrand et al., 2007; Sanz‐Sáez, Erice, Aguirreolea, Muñoz, et al., 2012; Table 3). For example, while lignin content was found to increase in response to e[CO_2_] when alfalfa was inoculated with one strain of *Sinorhizobium meliloti*, this was not the case with the two others assessed (Sanz‐Sáez, Erice, Aguirreolea, Muñoz, et al., 2012). Conversely, lignin concentrations have been found to decrease substantially in response to e[CO_2_] in mycorrhizal, but not non‐mycorrhizal, alfalfa plants (Baslam, Erice, & Goicoechea, 2012). Interestingly, despite the inconsistencies with respect to cell wall composition in legumes grown under e[CO_2_], in vitro digestibility (a measure of dry matter digestibility obtained through the incubation of plant samples with rumen fluid or enzyme preparations) has been found to remain unchanged at e[CO_2_] in the few studies where it has been assessed (Bertrand et al., 2007; Sanz‐Sáez, Erice, Aguirreolea, Muñoz, et al., 2012).

In addition to C:N ratio and digestibility, micronutrient levels can also play an important role in forage quality. While particular levels of micronutrients are required for plant growth itself, the presence of these elements in forage vegetative tissues is also of importance for animals that feed upon the plants, which means that sufficiency levels for both plants and animals need to be considered. For example, while boron (B) has not been shown to be essential in animals, legumes typically require more for growth and development than other plant species. Conversely, while copper (Cu), iron (Fe), manganese (Mn), zinc (Zn), and selenium (Se) levels are generally sufficient for optimum leguminous forage crop yields, the amounts present within forage tissues are frequently not adequate to meet the needs of livestock, and thus, supplementation can be required (Gupta, Kening, & Siyuan, 2008). Comparatively, little is known regarding the effect of e[CO_2_] on essential nutrient levels in leguminous forages, and of the few studies that have been carried out, results have been conflicting (e.g., Manderscheid et al., 1997; Pal et al., 2004). However, it appears that patterns of change for micronutrients tend to be similar between foliar and seed tissues in plants overall (Loladze, 2014), which suggests that findings for leguminous seeds in this context (see next section) may also hold true in forage crops and that responses may vary depending on the particular mineral.

### 4.2 Seed quality

Reductions in nitrogen concentration within photosynthetic tissues under e[CO_2_] have generally been found to correlate with decreased protein levels in seeds from C_3_ plants. Given the potential of legumes for less pronounced reductions in leaf nitrogen concentrations under e[CO_2_] compared to non‐N_2_‐fixing C_3_ plants, it is therefore not surprising that declines in seed nitrogen concentrations in legumes under e[CO_2_] also tend to be absent or smaller than in their non‐leguminous counterparts (Jablonski, Wang, & Curtis, 2002). Indeed, only 0%–5% decreases in seed nitrogen and/or protein concentrations are typically observed under e[CO_2_] for legumes such as soybean, lentil, kidney bean, and pea (Bellaloui et al., 2016; Bourgault et al., 2016; Bourgault et al., 2017; Jablonski, Wang, & Curtis, 2002; Köhler et al., 2019; Li et al., 2018; Thomas et al., 2009; Table 4), compared to the 10%–15% reduction in seed protein levels seen in other field‐grown C_3_ crops (Jablonski, Wang, & Curtis, 2002; Taub, Miller, & Allen, 2008). This is not unexpected seeing as legume seeds acquire the majority of their nitrogen through remobilization from leaf tissues during the early stages of seed filling (Ortez et al., 2019; Schiltz et al., 2005). However, this smaller impact on seed nitrogen levels is not always the case, and in chickpea, for example, greater reductions in seed protein concentration (between 8.4% and 10.2%) have been observed (Saha, Chakraborty, Sehgal, & Pal, 2015). In this particular instance, such a finding may be attributable to the fact that chickpea has a low capacity for N_2_‐fixation compared to other legumes (López‐Bellido et al., 2011). Furthermore, a high leaf nitrogen concentration at the flowering stage has not always been found to be a good indicator of a legume’s capacity to maintain seed protein levels under e[CO_2_] overall (Bourgault et al., 2016), and e[CO_2_] has been found to decrease the partitioning of nitrogen from vegetative tissues to seeds in at least certain cultivars of soybean (Li et al., 2017). In line with this, species‐ and cultivar‐specific differences involving not only the capacity for N_2_‐fixation, but also the ability to maintain nitrogen concentration specifically in seeds under e[CO_2_], have been suggested to play a role in such differential responses (Bourgault et al., 2016; Lam et al., 2012; Li et al., 2017).

**Table 4:** Examples of effects of long‐term^a^ e[CO_2_] on the quality of a selection of grain legumes

Legume	No.	Foliar [N]	Seed [N] or [protein]	Seed [oil]	Seed nutrients
Lentil	20^F^	4% average ↓ across cultivars	2% average ↓ across cultivars	ND	ND
	21^F^	ND	ND	ND	↓ in Zn and S no change in Fe, P, Ca, K, Na, Cu, Mg, or Mn
	22^F^	no change	3% ↑ or no change depending on cultivar	ND	ND
Faba bean	23^F^	ND	ND	ND	↓ in Ca, Mg, S, Fe and Zn no change in P, Na, Cu, Mn, or K
Soybean	25^OP^	ND	2%–6% ↓ in mature seeds depending on cultivar	9%–13% ↑ in [oil] in mature seeds depending on cultivar ↑ in stearic acid in mature seeds variable effects on oleic and linoleic acid depending on cultivar	↓ in Fe in mature seeds ↑ or no change in Mg, S, and Ca depending on cultivar ↑ or ↓ in Mn and Cu depending on cultivar ↓ or no change in Zn depending on cultivar no change in P or K
	27^OP^	5% average ↓ across 19 cultivars 3% average ↑ across 5 cultivars	ND	ND	ND
	30^OF^	ND	no change	no change	ND
	31^F^	no change	no change	ND	ND
	32^GC^	ND	5% ↓ across cultivars	5%–7% ↑ in [oil] depending on cultivar ↑ oleic acid, ↓ linolenic acid	↓ in P, K, Fe, and B ↓ or no change in Zn depending on cultivar no change in Mg, Cu, or Mn
	35^OP^	ND	no change at maturity	no change in [oil] at maturity ↓ in linolenic acid at maturity	ND
	36^F^	ND	no change	no change	↓ in Fe and Zn
Mung bean	38^OF^	ND	ND	↑ in omega‐3 FAs ↓ in palmitic and omega‐6 FAs	ND
Field pea	39^F^	4%–5% average ↓ across cultivars depending on growth stage	3% average ↓ across cultivars	ND	ND
	40^F^	ND	no change	ND	↓ in Zn in one soil type and year no change in P, K, Ca, Fe, Mn, Mg, or Cu
Chickpea	42^OF^	ND	8%–10% ↓ depending on year	30%–32% ↓ depending on year	↓ in S, K, Ca, and Zn no change in P, Mg, Cu, Fe, Mn, or B

Note

Results are from studies carried out at ambient temperatures without intentional drought treatments.

Abbreviations: B, boron; Ca, calcium; Cu, copper; Fe, iron; ^F,^ free‐air CO_2_ enrichment; FA, fatty acid; ^GC,^ growth cabinet; ^GH,^ greenhouse; K, potassium; Mg, magnesium; Mn, manganese; N, nitrogen; Na, sodium; ND, no data; No., number assigned to study in Table S1; ^OF,^ open‐top chamber sown in field; ^OP,^ open‐top chamber in pots; P, phosphorus; S, sulfur; and Zn, zinc.

^a^ Treated for >3 weeks.

While seed protein concentration is certainly an important consideration in terms of nutritional quality, as is the case in forages, essential nutrients also comprise a vital component. Indeed, it has been estimated that upwards of 2 billion people globally are currently deficient in one or more nutrients, with approximately 1.5 billion being deficient in Zn (Smith & Myers, 2018) and approximately 2 billion lacking in Fe (Viteri, 1998). Unfortunately, in legumes, Fe and Zn concentrations have been found to decline under e[CO_2_] in lentil, faba bean, field pea, and soybean (Li et al., 2018; Myers et al., 2014; Parvin et al., 2019), which could exacerbate micronutrient deficiencies in the future. In contrast, alterations in the levels of P, S, Mg, and K under long‐term e[CO_2_] have been extremely inconsistent across studies, cultivars, and species, exhibiting increases, decreases, or no changes compared to ambient [CO_2_] (e.g., Hao et al., 2016; Li et al., 2018; Parvin et al., 2019; Table 4).

The mechanisms responsible for changes in mineral concentrations under e[CO_2_] remain to be elucidated; however, it has been speculated that reductions in stomatal conductance seen under these conditions may decrease the uptake of nutrients that depend upon transpiration‐driven mass flow (Houshmandfar et al., 2018; McGrath & Lobell, 2013). Alternatively, it has also been proposed that the stimulation of seed yield often seen under e[CO_2_] may lead to a concomitant “dilution effect” with respect to seed nutrient concentration (Poorter et al., 1997), although this does not appear to be the case for all minerals (Parvin et al., 2019). Whatever the mechanism, it appears that e[CO_2_] can have both negative and, in certain instances, positive effects on micronutrient levels in legume seeds, and further research will be required to better understand such responses.

In addition to the value of legumes as an important source of protein, certain species such as soybean are utilized as an oilseed crop and are also economically vital to the agricultural sector (Ainsworth et al., 2012). There is a paucity of information regarding the effect of e[CO_2_] on seed oil content and composition in legumes, although results from the handful of studies that have been carried out are variable (Table 4). While certain studies have found no change (Köhler et al., 2019; Palacios et al., 2019; Rogers, Cure, & Smith, 1986; Taub, Miller, & Allen, 2008; Thomas et al., 2003) or an increase (Bellaloui et al., 2016; Hao et al., 2014; Heagle, Miller, & Pursley, 1998; Li et al., 2018) in legume seed oil content when grown under CO_2_ enrichment, others have observed a reduction (Heagle, Miller, & Pursley, 1998; Saha, Chakraborty, et al., 2015). Furthermore, while the fatty acid composition of legume seed oil, which has important implications for downstream applications and health properties, has been found to be significantly altered under e[CO_2_] in a subset of studies (Hao et al., 2014; Heagle, Miller, & Pursley, 1998; Li et al., 2018), this has not always been the case (Rogers, Cure, & Smith, 1986; Thomas et al., 2003). Where differences have been noted, results have again been erratic, with both increases and decreases in the proportion of linolenic acid observed under e[CO_2_] in soybean (Bellaloui et al., 2016; Hao et al., 2014; Li et al., 2018; Palacios et al., 2019), and increases in the percentage of omega‐3 fatty acids at the expense of omega‐6 fatty acids in mung bean (Dey et al., 2017), for example. As with other responses to e[CO_2_], it is likely that cultivar‐ and species‐specific differences, as well as growth and environmental conditions, may play a role in these inconsistencies (Li et al., 2018).

Soybeans also contain large amounts of carbon‐based isoflavones, which are polyphenolic secondary metabolites considered to have many health benefits, including the inhibition of ovarian and colon cancer cell growth (Chang et al., 2007; MacDonald et al., 2005) and the reduction of serum low‐density lipoprotein cholesterol levels (Taku et al., 2007). Although few studies have been carried out assessing the effects of long‐term e[CO_2_] exposure on isoflavone levels, it seems likely that increases in the amount of carbon fixed through photosynthesis under e[CO_2_] would drive increased production of this biomolecule (as is the case for condensed tannins). Indeed, a small number of studies have provided evidence for this, whereby significant increases in isoflavone concentrations have been found in soybean seeds under CO_2_ enrichment (Kim et al., 2005; Li et al., 2018).

## 5 EFFECT OF ELEVATED [CO_2_] ON STRESS RESPONSE

The production of reactive oxygen species (ROS), including superoxide (O_2_^−^) and hydrogen peroxide (H_2_O_2_), are an inevitable by‐product of aerobic metabolism (Mittler et al., 2011). These molecules are produced constitutively in plants at relatively low levels through photosynthesis, photorespiration, β‐oxidation of fatty acids, and the mitochondrial electron transport chain, for example (Apel & Hirt, 2004; Sharma et al., 2012). However, when exposed to abiotic and biotic stress conditions, ROS levels tend to increase rapidly, whereby they function as signal transduction molecules (Baxter, Mittler, & Suzuki, 2014). This leads to alterations in the redox state of regulatory proteins, as well as changes in gene expression and translation, that allow adaptation to stress conditions (Choudhury et al., 2016; Xia et al., 2015). While providing benefits in terms of tolerance to various forms of stress, above certain thresholds ROS can harm plant cells through their oxidation of membrane lipids, as well as the damage they incur to proteins, chlorophyll, and nucleic acids (Foyer & Harbinson, 1994). To diminish these deleterious effects, plants possess mechanisms to scavenge and de‐toxify excess ROS through an integrated system of enzymatic and non‐enzymatic antioxidants, which also tend to be up‐regulated under stress conditions (Das & Roychoudhury, 2014).

An overall “CO_2_‐protective effect” has been suggested to occur in various plant species, which could potentially protect against various types of stress at the whole plant growth level, as well as through reductions in oxidative damage (i.e., lipid peroxidation and protein oxidation) under e[CO_2_] (AbdElgawad et al., 2015; Clifford et al., 1993; Wang et al., 2018; Zinta et al., 2014). However, in practice, such a benefit does not appear to occur across different types of stress or species (e.g., Jifon & Wolfe, 2005; Prasad et al., 2002; Prasad, Allen, & Boote, 2010; Thomey et al., 2019; Ziska & Bunce, 1994). While the precise molecular mechanisms driving stress‐related responses under CO_2_ enrichment have yet to be unraveled, it may involve the diminished production of ROS typically observed under e[CO_2_], which potentially occurs as a result of increased carboxylation rates and a reduction in oxygenation/photorespiration (AbdElgawad et al., 2016). On one hand, reductions in ROS under e[CO_2_] may decrease perceived oxidative stress, as well as actual oxidative damage, which could explain the reduction in total antioxidant capacity that is often evident in legumes under these conditions (Aranjuelo et al., 2008; Gillespie, Rogers, & Ainsworth, 2011; Pritchard et al., 2000). However, it is also possible that a decrease in ROS, which also act as a signal to augment antioxidant defense systems as a means of priming plants to respond to additional stress exposure (Tausz et al., 2013), could also feasibly lead to reduced stress tolerance.

Alterations in the levels of phytohormones, which are used universally by plants to effect appropriate responses to both abiotic and biotic stress (Xia et al., 2015), are also commonly observed under long‐term exposure to e[CO_2_]. In particular, reductions in basal jasmonic acid (JA) and ethylene (ET) levels, along with the expression of related transcripts, have been encountered in some, but not all, soybean cultivars under e[CO_2_] (Casteel et al., 2008; Guo et al., 2014; Zavala et al., 2008). Conversely, concomitant increases in the antagonistic salicylic acid (SA)‐dependent pathway have been observed across multiple cultivars of both soybean and bean exposed to CO_2_ enrichment (Casteel et al., 2012; Mhamdi & Noctor, 2016). Since phytohormones exert their function in part through the activation of ROS production (Xia et al., 2015), it may be that an interplay between phytohormone and ROS levels is responsible, at least in part, for generalized alterations in stress response seen under e[CO_2_].

### 5.1 Abiotic stress resilience

**Figure 3:**
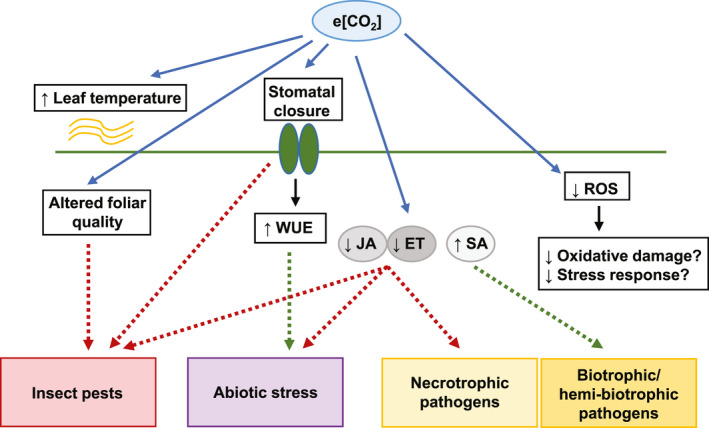
Possible effects of long‐term e[CO_2_] exposure on stress responses in legume species. Red discontinuous arrows indicate reduced tolerance compared to ambient [CO_2_], while green discontinuous arrows denote increased tolerance. ET, ethylene; JA, jasmonic acid; SA, salicylic acid; ROS, reactive oxygen species; WUE, water‐use efficiency; and Zn, zinc

While the climate change effects resulting from rising greenhouse gas emissions can have serious negative impacts on crop yields, the interactive impact of e[CO_2_] and these abiotic stress factors is only beginning to be deciphered. It has been suggested that legumes are particularly prone to reductions in ROS production and downstream oxidative stress effects in response to drought and elevated temperature under e[CO_2_] compared to ambient [CO_2_] (AbdElgawad et al., 2015). Although there is some divergence in opinion as to whether such effects will have positive or negative outcomes in terms of abiotic stress tolerance, the reductions in JA and ET typically seen under e[CO_2_] suggest that responses may be inferior overall due to the positive regulatory roles that these phytohormones normally play in salinity, drought, heat (JA‐related), and flooding (ET‐related) response (Kazan, 2015; Figure 3). However, studies carried out thus far suggest that the relationships between CO_2_ levels and abiotic stress in terms of plant response and resilience are very complex (Table 5), and large gaps remain in our understanding of these interactions.

**Table 5:** Examples of effects of high temperature and drought in combination with long‐term^a^ e[CO_2_] on a selection of legumes

Legume	No.	Photosynthesis (A_growth_, acclimation)	Biomass	Seed yield	Quality
Forage					
Alfalfa	4^GH^	ND	no change at atemp with any rhizobial strain ↑ or no change at etemp depending on rhizobial strain	ND	similar changes in NDF and ADF at atemp and etemp no change in IVDMD at any temperature ↑ or no change in lignin at atemp, ↓ or no change at etemp, depending on rhizobial strain ↓ or no change at atemp and etemp depending on rhizobial strain no change in [TSS] at any temp
	5^TP^	no change in magnitude of acclimation with etemp or drought	ND	ND	foliar [N] ↓ under atemp, etemp and well‐watered conditions, no change under etemp + drought no change in [TSP] under atemp, well‐watered or drought conditions, ↓ at etemp ↑ at atemp and etemp + drought, no change under etemp or drought
	7^TP^	acclimation at atemp and etemp, well‐watered and drought, prior to cutting 1 month after cutting, acclimation only at etemp, not drought, well‐watered or atemp	ND	ND	after 1 month of regrowth, no change in [TSS] under well‐watered and atemp, no change under drought at any temperature, ↓ under etemp ↑ in [TSP] under well‐watered and atemp, no change at etemp, ↓ under drought at all temperatures
	8^GC^	↑ in A_growth_ at 15°C and 20°C, no change at 25°C and 30°C acclimation only observed at 30°C	smaller ↑ in leaf and stem DW ↑ as temperature increased root DW ↑ at 15°C, 20°C and 25°C, no change at 30°C	ND	↓ in [TSP] at 15°C, 20°C and 30°C, no change at 25°C
Subclover	14^TF^	ND	greater ↑ in shoot DW at etemp than atemp	ND	ND
	15^TF^	ND	ND	ND	[NSC] ↑ at atemp and etemp similar ↓ in shoot [N] at atemp and etemp
Grain					
Lentil	21^F^	ND	ND	smaller ↑ in dry year than wet year	greater ↓ in P, S, Fe, and Zn in dry year than wet year ↓ in Ca in dry season, no change in wet season
	22^F^	no consistent difference between wet and dry years	smaller ↑ in ABG DW in dry year than wet year	↑ in wet year, no change in dry year	grain [N] ↓ in dry season, no change in wet season
Faba bean	23^F^	ND	ND	smaller ↑ in dry year than wet year	greater ↓ in P, S, Fe, and Zn in dry year than wet year
Soybean	32^GC^	ND	ND	ND	↓ in seed [protein] at atemp, no change at etemp ↑ in seed [oil] at atemp temperature, no change or ↑ at etemp depending on cultivar changes in grain nutrient levels between atemp and etemp varied with cultivar
	34^b^ ^,OP^	greater ↑ in A_growth_ under drought than well‐watered at full bloom A_growth_ ↑ at seed filling stage under well‐watered conditions, no change under drought	15% and 23% average ↑ in ABG biomass across years under well‐watered and drought conditions, respectively	13% and 30% average ↑ across years under well‐watered and drought conditions, respectively	ND
	35^OP^	ND	overall smaller ↑ in leaf and stem DW at etemp than at atemp	no significant change under atemp or etemp	no change in grain [protein] at atemp or etemp at maturity no change in grain [oil] at atemp or etemp at maturity
	36^F^	ND	no substantial difference between atemp and etemp	↑ under both atemp and etemp, but yields were substantially less under etemp	no consistent change in grain [protein] or [oil] at atemp or etemp
Kidney bean	46^b^ ^,GH^	at atemp, A_growth_ ↑ at all growth stages at etemp, A_growth_ ↑ during early growth stages, but no change by 54 DAP higher levels of acclimation at etemp than atemp	greater ↑ in total vegetative DW at atemp than etemp at maturity	↑ at atemp, ↓ at etemp	ND
	47^b^ ^,CF^	smaller ↑ in A_growth_ at higher temperatures	↑ in total DW similar between 28/18ºC and 37/27ºC, no change at 40/30ºC	smaller ↑ as temperature increased	ND
Groundnut					
Peanut	51^GHF^	ND	greater ↑ in ABG DW under drought than well‐watered conditions	greater ↑ under drought than well‐watered conditions	ND

Abbreviations: ABG, aboveground; ADF, acid detergent fiber; A_growth_, net photosynthetic rate at saturating (or near‐saturating) light under growth [CO_2_], atemp, ambient temperature; Ca, calcium; DAP, days after planting; DW, dry weight; etemp, elevated temperature; ^F,^ free‐air CO_2_ enrichment; Fe, iron; ^GC,^ growth cabinet; ^GH,^ greenhouse; ^GHF,^ greenhouse sown in field; IVDMD, in vitro dry matter digestibility; K, potassium; N, nitrogen; ND, no data; NDF, neutral detergent fiber; No., number assigned to study in Table S1; NSC, foliar non‐structural carbohydrates; ^OP,^ open‐top field chamber in pots; ^TF,^ temperature gradient enclosure sown in field; ^TP,^ temperature gradient enclosure in pots; P, phosphorus; S, sulfur; ^TGF,^ temperature gradient in field; TSP, foliar total soluble protein; TSS, foliar total soluble sugars; and Zn, zinc.

^a^ Treated for >3 weeks.

^b^ At least a subset of plants received exogenous N throughout experiment.

In all plant species, including legumes, e[CO_2_] tends to reduce stomatal conductance, which leads to lower levels of leaf transpiration and hence increased water‐use efficiency (WUE; Leakey et al., 2009). Such an improvement in WUE has the potential to lead to soil water conservation that could be utilized later in the growing season (Niklaus, Spinnler, & Körner, 1998). This phenomenon could theoretically lend to greater relative increases in yield under drought in the presence of e[CO_2_] than ambient [CO_2_] since the time required to reach a particular water stress would be increased. In line with this, enhancements in performance under drought conditions were observed under CO_2_ enrichment in greenhouse experiments with peanut (Clifford et al., 1993) and open‐top field chambers with soybean (Rogers, Cure, & Smith, 1986).

However, increased soil water conservation under e[CO_2_] does not appear to always be the case in practice (e.g., Gray et al., 2016; Parvin et al., 2018; Saha et al., 2011), and early increases in root and shoot biomass seen under e[CO_2_] may outweigh reductions in transpiration on a leaf‐level basis. If this is indeed the case, growth under e[CO_2_] would instead lead to increased water usage at the whole plant level and thus an earlier onset of drought. This could cause more intense water deficits during developmental stages, which are particularly harmful, diminishing any potential gains that could be incurred through CO_2_ enrichment (Bourgault et al., 2017). Furthermore, since N_2_‐fixation is particularly sensitive to water stress (Serraj, Sinclair, & Allen, 1998), nitrogen dilution in response to e[CO_2_] may also occur more readily under drought than well‐watered conditions, leading to a higher propensity for photosynthetic acclimation, less substantial yield gains, and reductions in quality (Ainsworth et al., 2002). Correspondingly, in the majority of studies, photosynthetic and/or yield gains in response to e[CO_2_] have been found to be reduced or lacking in legumes without an adequate water supply compared to those that were well‐watered (e.g., Aranjuelo et al., 2009; Gray et al., 2016; Parvin et al., 2018). In addition, small but significant decreases in legume seed protein concentration have been observed in field pea (Bourgault et al., 2016), lentil (Parvin et al., 2018) and soybean (Gray et al., 2013) under e[CO_2_] in arid conditions, while no changes or even increases were observed in high rainfall environments in soybean and lentil, respectively (Gray et al., 2013; Parvin et al., 2018).

With respect to rising temperatures, effects on legume production are likely to depend upon the geographical region, with temperature increases in more temperate climates enhancing e[CO_2_]‐derived yield gains and those in already warm areas leading to a decline in the stimulatory effects of CO_2_ (Kromdijk & Long, 2017). Indeed, when temperatures are above an optimal level, legumes tend to be more susceptible to photosynthetic acclimation under e[CO_2_], even when provided with exogenous nutrients (Sanz‐Sáez, Erice, Aguirreolea, Irigoyen, Sánchez‐Díaz, 2012; Ziska & Bunce, 1994). Such a phenomenon tends to be associated with an absence or reduction in yield enhancements (Bourgault et al., 2018; Heinemann et al., 2006; Prasad et al., 2002; Ziska & Bunce, 1994). As is the case with drought stress under e[CO_2_], these findings likely derive, at least in part, from the decreases in N_2_‐fixation often seen under high temperatures (e.g., Aranjuelo, Irigoyen, & Sánchez‐Díaz, 2007; Hungria & Kaschuk, 2014), although effects may also be dependent upon the strain of symbiont utilized (Sanz‐Sáez, Erice, Aguirreolea, Irigoyen, et al., 2012).

Reproductive development in plants, including the production of flowers, pollination, and seed filling, tends to be more sensitive to elevated temperatures than vegetative growth or photosynthesis. In line with this, temperatures that are beneficial for biomass production and photosynthesis often prove harmful for reproductive growth, leading to reductions in yield (Prasad et al., 2002). Therefore, as with drought stress, the timing of heat stress can have a substantial impact on its interactive effect with e[CO_2_], with high temperatures and/or heat waves incurred during reproductive growth tending to have the most deleterious outcomes (Jifon & Wolfe, 2005; Thomey et al., 2019). In addition, the threshold temperature for seed set has been found to be reduced under e[CO_2_], which indicates that yield losses due to high temperatures will likely increase with rising CO_2_ (Prasad et al., 2002), at least in sub‐tropical and tropical regions where average temperatures are already at the optimum for seed production in legumes such as soybean, cowpea (*Vigna unguiculata*), and peanut (Prasad, Allen, & Boote, 2010). While the mechanism(s) driving such a phenomenon has yet to be elucidated, a lower tolerance for high temperatures may be related to the finding that long‐term exposure to e[CO_2_] increases leaf temperatures in soybean (Valle et al., 1985) and kidney bean (Prasad et al., 2002) by approximately 1.5–2°C compared to plants grown under ambient [CO_2_]. This likely occurs due to decreased transpirational cooling as a result of reductions in stomatal conductance (Ruiz‐Vera et al., 2013).

Micronutrient concentrations under e[CO_2_] have also been found to be differentially affected in legumes when exposed to drought and elevated temperatures. For example, greater decreases in concentrations have been observed under e[CO_2_] when lentil, faba bean, field pea, and soybean were grown in dry rather than wet conditions (Parvin et al., 2019). While the opposite may hold true for small temperature increases, as has been found in soybean under CO_2_ enrichment (+2.7°C/+3.4°C day/night; Köhler et al., 2019), it is not known as of yet whether this will also be the case with more extreme temperature variations. Therefore, further direct experimental investigations of the interactive effects of CO_2_ enrichment and other environmental stresses on mineral concentrations will be necessary to determine what the precise impact of e[CO_2_] will be on human and livestock nutrition in the future (Parvin et al., 2019).

While relatively few studies aimed at evaluating the combined effects of drought, temperature and e[CO_2_] have been carried out on leguminous species as of yet, existing data from a growing number of controlled environment and FACE studies suggest that interactions are highly multifaceted (e.g., AbdElgawad et al., 2015; Bishop, Leakey, & Ainsworth, 2014; Delahunty et al., 2018). Since optimum temperatures and water availability for photosynthesis, vegetative growth, and reproductive development can differ quite drastically among species (Prasad, Allen, & Boote, 2010), precise interactions will likely be species‐dependent. In addition, the severity and timing of stress conditions, genotype, nutritional factors, soil type, and geographical region will almost certainly also play a substantial role in the particular response elicited.

### 5.2 Disease resistance

Plant disease expression is dependent on a susceptible host plant, virulent pathogen, and environmental conditions that can influence pathogen infection. Changes in temperature, precipitation, and atmospheric conditions are the most frequently studied in epidemiological models of pathogen expansion and disease expression under climate change scenarios (reviewed by Bebber, 2015; Coakley, Scherm, & Chakraborty, 1999). However, there are only a very limited number of studies describing changes in disease expression that are directly related to e[CO_2_] for legume plants as of yet.

Although responses to e[CO_2_] vary quite substantially with the host–pathogen system, the general trend is that there are divergent effects of e[CO_2_] on biotrophic versus necrotrophic pathogens. This finding is linked to the antagonism between the SA and JA/ET pathways (Casteel et al., 2012; Kazan, 2018; Mhamdi & Noctor, 2016; Zhang et al., 2015; Zhou et al., 2019; Figure 3), with SA playing a key role in local and systemic responses to biotrophic and hemi‐biotrophic pathogens and JA/ET providing a central function in response to necrotrophic pathogens (Glazebrook, 2005). The constitutive up‐regulation of the SA pathway seen in legumes grown under e[CO_2_] could therefore theoretically lead to increased resistance to biotrophs under these growth conditions. Correspondingly, the severity of downy mildew, caused by the biotrophic pathogen *Peronospora manshurica*, was found to be consistently reduced in soybean under e[CO_2_] in three years of a FACE study, despite annual differences in temperature and rainfall (Eastburn et al., 2010). However, the up‐regulation of the SA pathway under CO_2_ enrichment has not always been found to be the case in non‐legumes such as barley (*Hordeum vulgare*) (Mhamdi & Noctor, 2016) and tobacco (*Nicotiana tabacum*) (Matros et al., 2006), suggesting that this may be a species‐specific response. Further research will be required to determine whether the response of the SA pathway under e[CO_2_] also varies among legumes.

Conversely, declines in the JA/ET pathway under e[CO_2_] (Casteel et al., 2008; Casteel et al., 2012) could conceivably lead to decreased resistance to necrotrophic pathogens. In line with this, while e[CO_2_] treatment had no significant effect on the incidence of Septoria brown spot of soybean caused by the necrotrophic pathogen *Septoria glycines*, there was a significant increase in disease severity in all 3 years of the study (Eastburn et al., 2010). In the case of hemi‐biotrophic pathogens, responses may be less predictable. For example, an enhancement in physiological resistance to anthracnose (*Colletotrichum gloeosporioides*; hemi‐biotroph) has been observed under e[CO_2_] in the perennial pasture legume *Stylosanthes scabra* in controlled environmental growth conditions; however, an increase in severity was evident when infection took place following the transfer of plants to field conditions at ambient [CO_2_] (Pangga, Chakraborty, & Yates, 2004). It remains to be determined what effect CO_2_ enrichment in field conditions would have on the susceptibility of a leguminous species to hemi‐biotrophic pathogens. Although nodulation was not assessed in any of these studies, nodulation‐related processes can interact with defense responses through changes in hormone biosynthesis and signaling pathways that may in turn alter the outcome of pathogen infection (Berrabah et al., 2018; Rey & Jacquet, 2018). Therefore, comparisons of pathogen infection in the absence and presence of rhizobium should be carried out in future studies in which disease responses under e[CO_2_] are examined in order to expand our understanding of these interactions.

Phytohormone‐related alterations in response to e[CO_2_] are not the only factor involved in changes to disease susceptibility, and it has been proposed that any enhancements in disease resistance under CO_2_ enrichment may be offset by a larger and denser canopy under field conditions, which provides a more favorable microclimate for disease development and allows the trapping of more spores (Pangga, Chakraborth, & Yates, 2004; Pangga, Hanan, & Chakraborty, 2011). However, additional field trials with legume plants under e[CO_2_] conditions are needed to confirm this with any certainty. Several other plant physiological changes that occur in response to e[CO_2_] could also potentially influence host resistance. For example, the promotion of net photosynthesis observed under CO_2_ enrichment may allow increased partitioning of resources into the production of carbon‐based defense compounds, such as phytoalexins (Braga et al., 2006; Kretzschmar et al., 2009; Mhamdi & Noctor, 2016), and augmented foliar carbohydrate concentrations could also potentially alter the growth of at least certain biotrophic pathogens (reviewed by Manning & Tiedemann, 1995). Improvements in resistance could also feasibly be elicited through the production of elevated quantities and altered compositions of leaf surface waxes, as well as increases in the number of epidermal cell layers (reviewed by Kazan, 2018). In addition, the reductions in stomatal conductance observed under e[CO_2_] have been implicated in increased resistance to stomatal invading pathogens such as bacteria and many biotrophs, as these pathogens would encounter fewer infection sites and lower humidity on leaf surfaces due to stomatal closure (Eastburn, McElrone, & Bilgin, 2011; Li et al., 2015).

Given that the effects of e[CO_2_] on disease expression often appear to be pathogen‐/host‐specific, and that very few studies have been performed on defense responses and associated diseases of legume host–pathogen systems under e[CO_2_], further investigations are needed to clarify the changing risk of plant diseases on important legume crops. Furthermore, given the integral role of temperature, precipitation, and canopy microclimatic variables on disease expression, the interaction of these environmental conditions with e[CO_2_] will also require additional study to develop epidemiological models under climate change for diseases of legumes.

### 5.3 Pest tolerance

Crop damage sustained from insect herbivores is already a major challenge for the production of many crops, and it is recognized that future atmospheric and climatic conditions could worsen such issues (Gregory et al., 2009). While many factors related to climate change have the potential to exacerbate pest infestations, e[CO_2_] itself has often been shown to decrease plant tolerance through alterations in particular quality traits, defense molecules, and/or antioxidant enzyme activities (e.g., Martin & Johnson, 2011; Stiling & Cornelissen, 2007; Zavala et al., 2008). However, insects within different feeding guilds may respond differently to changes in plant primary and/or secondary metabolism under e[CO_2_], and the mechanisms behind these disparities have yet to be fully unraveled.

The majority of studies aiming to decipher the effect of e[CO_2_] on insect pests of legumes have focused on chewing insects, including lepidopteran and coleopteran species, whereby susceptibility to these herbivores has been found to increase under e[CO_2_] (e.g., Yifei et al., 2018; Zavala et al., 2008; Figure 3). Such a response has been suggested to derive, at least in part, from the increase in foliar C:N ratios commonly encountered under these growth conditions in many plant species. Since nitrogen is the most limiting resource for phytophagous insects (Mattson, 1980), the decrease in leaf nitrogen concentrations commonly seen under e[CO_2_] can result in reduced larval and pupal weights, prolonged larval and pupal life spans, and decreased relative growth rates (e.g., O’Neill et al., 2008; Yifei et al., 2018). However, herbivorous insects, and particularly lepidopteran insects, compensate for this by increasing their total consumption, leading to higher levels of herbivore damage (e.g., Srinivasa Rao et al., 2009; Srinivasa Rao et al., 2015; Srivastava et al., 2002; Yifei et al., 2018). While this may certainly be the case in non‐leguminous C_3_ plants, one would expect this to have less of an impact in legumes due to the smaller reductions in foliar nitrogen typically seen under e[CO_2_] in these species (Karowe, 2007). As such, where increased susceptibility to chewing insects is observed in legumes, other factors may also be key.

It has been suggested that increases in foliar non‐structural carbohydrate levels may also act as a phagostimulant in the case of coleopteran insects such as the Japanese beetle (*Popillia japonica*) and Mexican bean beetle (*Epilachna varivestis* Mulsant.), leading to a preference for legume foliage grown at e[CO_2_] over ambient [CO_2_] (Hamilton et al., 2005). While sugars may stimulate feeding in Japanese beetles, this has not always been found to be the case (O’Neill et al., 2008; Zavala et al., 2008), and instead, it has been proposed that alterations in plant chemical defenses may play a greater role. For example, genes related to JA‐ and ET‐mediated defense signaling, which are important defense pathways in the context of pest tolerance, have been shown to be down‐regulated under e[CO_2_] both constitutively and under induction by herbivory (Casteel et al., 2008; Casteel et al., 2012; Zavala et al., 2008). This could function to suppress the capacity to mount an effective defense against herbivorous insect pests, thus increasing susceptibility. Furthermore, alterations in JA‐ and ET‐signaling pathways under e[CO_2_] have also been found to correlate with a reduction in the production of cysteine proteinase inhibitors, which act as specific deterrents to coleopteran herbivores (Zavala et al., 2008). In line with this, gut cysteine proteinase activity was found to be higher in Japanese beetles and Western corn rootworm (*Diabrotica virgifera virgifera*) that consumed the foliage of soybean grown under CO_2_ enrichment than those fed foliage grown under ambient [CO_2_], which led to increases in their growth and development under e[CO_2_] (Zavala et al., 2008).

Phloem‐feeding aphids, which comprise one of the most detrimental pests to crop production globally, may also become more problematic under future atmospheric conditions (Figure 3). Indeed, certain aphid species themselves display enhanced survival, fecundity, and abundance under CO_2_ enrichment (Robinson, Ryan & Newman, 2012), which could prove problematic. In legumes, the pea aphid (*Acyrthosiphon pisum* (Harris)) can be a particular challenge as it exhibits a very wide geographical distribution and feeds on an extensive range of species (Blackman & Eastop, 2000). As is the case with chewing insects, down‐regulation of the JA/ET‐signaling pathway has been found to occur in *M. truncatula* during pea aphid infestation under e[CO_2_] compared to ambient [CO_2_] (Guo et al., 2014), which may contribute to the increases in susceptibility that are typically observed in this species at elevated CO_2_ levels (Guo et al., 2014). Aphid‐infested *M. truncatula* plants have also been found to exhibit lower activities of various antioxidant and secondary metabolism enzymes under e[CO_2_] compared to ambient [CO_2_], as well as elevated leaf temperatures and reduced stomatal apertures, which could also play a role in increased susceptibility under these conditions (Guo et al., 2014; O’Neill et al., 2011; Sun, Guo, & Ge, 2016). Increases in the availability of non‐essential amino acids have also been observed in *M. truncatula* under e[CO_2_], which may enhance their nutritional quality and thus promote the growth of pea aphid populations (Guo, Sun, Li, Tong, et al., 2013).

Although relatively few studies have been carried out thus far regarding the effect of CO_2_ levels on aphid–legume interactions, it is clear that responses are highly genotype‐specific. It has been suggested that quantitative and qualitative alterations in foliar amino acids under e[CO_2_] may play a role in the susceptibility or resistance of particular genotypes under these conditions, although direct evidence of causality is still lacking (Guo, Sun, Li, Tong, et al., 2013; Guo et al., 2014; Johnson, Ryals, & Karley, 2014). Differential genotype‐specific responses under e[CO_2_] have also been attributed to the presence or lack of resistance (R) genes targeting particular aphid biotypes, along with associated effector‐triggered immunity (ETI) responses. For example, *M. truncatula* genotypes possessing an R gene have been found to increase JA signaling and exhibit increased resistance to pea aphid under e[CO_2_], while genotypes lacking an R gene displayed reductions in JA signaling and decreased resistance to infestations (Sun et al., 2018). However, this does not always appear to be the case, since R gene‐dependent resistance to the European large raspberry aphid (*Amphorophora idaei*) has been found to decrease in red raspberry (*Rubus idaeus*) grown under e[CO_2_] (Martin & Johnson, 2011). These findings imply that different cultivars/species may exhibit distinct alterations in their susceptibility to aphid infestations under a future of rising CO_2_ levels, which will be an important consideration for downstream breeding endeavors.

Taken together, it seems that overall, plant responses to e[CO_2_] will likely lead to altered interactions between plants and insects, resulting in more acute and frequent outbreaks of invasive insect pests. Genotypic differences in such responses to particular insect pests suggest that the genetic potential exists in at least certain legumes for improvement of these outcomes in the future. However, further research in this area will be imperative to better our understanding of both the responses of legumes to insect pests under e[CO_2_] and the precise mechanisms driving these responses in order for such improvements to be realized.

## 6 CONCLUSIONS

Given the importance of leguminous species as sources of high quality food and feed, increasing both vegetative biomass and seed yields, while maintaining quality, will be crucial to food security as our global population soars in coming years. Since substantial expansions in cropping area will not likely be an option, enhancing yield gains using both conventional and biotechnological breeding approaches will be critical to achieving this goal. The development and commercialization of new crop cultivars can take decades; therefore, these new varieties will need to be optimized now for the vastly different atmospheric conditions that will be evident in coming years.

While much research is dedicated to the effects of climate change on crop production within a weather‐related framework (i.e., increasing temperatures, drought, flooding), the direct roles of greenhouse gases such as CO_2_ are often overlooked. To date, findings regarding the effects of e[CO_2_] on the yield and quality of leguminous forages and pulse/soybean seed have been inconsistent for the most part. This likely results from the complexity of the responses themselves, as well as the vast number of interacting elements, including genotype/species‐specificity, rhizobial strain, CO_2_ treatment level, rooting volume, water/nutrient availability, soil type, irradiation level, and stress incidence. Therefore, it currently remains uncertain how future atmospheric conditions will actually impact legume yield and quality. While it seems that legumes may exhibit superior yield benefits and reduced protein decreases under e[CO_2_] compared to non‐N_2_‐fixing plants, in many instances they still do not maximize yield gains or completely prevent nitrogen depletion when grown at elevated CO_2_ levels.

Indeed, evidence is mounting to indicate that under limiting conditions, which may well be encountered rather frequently in field conditions in a future of climate change, it is probable that overall declines in many of these parameters will instead be the reality. Such a consequence would have a considerable negative effect on food security, as well as human and animal health. While teasing apart the intricate process of e[CO_2_] response in legumes will certainly not be simple, furthering our knowledge concerning e[CO_2_]‐related outcomes in legumes with respect to yields, quality, and ability to withstand other forms of abiotic and biotic stress will be a requisite for the breeding of new high‐yielding legume varieties for the future.

## CONFLICT OF INTEREST

The authors declare no conflict of interest.

[Correction added on 24 May 2021, after first online publication: Conflict of Interest statement added to provide full transparency.]

## AUTHOR CONTRIBUTIONS

SDS conceived the topic of the article. SDS, SC, RYS, US, GC, and SNA all contributed to the writing of the manuscript. All authors read and approved the final version of the manuscript.
