# Supplementary material for: Potential effects of a high CO2 future on leguminous species
Source: Plant Environ Interact. 2020 Apr 24;1(2):67–94. doi: 10.1002/pei3.10009 (PMC10168062; doi:10.1002/pei3.10009)
Supplement: Supplementary file 1 [file PEI3-1-67-s001.docx]

**Supplemental Table 1** Growth conditions used in a selection of studies where photosynthesis and growth/yield were assessed under long-term^†^ e[CO_2_] in legume species

| **Species** | **Cultivar** | **Symbiont** | **Growth** | **Pot size** | **Light (µmol m^-2^ s^-1^** | **Temp (°C)**  **(D/N)** | **Hum**  **(%)** | **Phot (h) (D/N)** | **[CO_2_]**  **(ppm)** | **Ref** | **No.** |
| --- | --- | --- | --- | --- | --- | --- | --- | --- | --- | --- | --- |
| Forages |  |  |  |  |  |  |  |  |  |  |  |
| *Medicago sativa*  (alfalfa) | Aragón | *Sm* 102F78 | GH | 2L, 4/pot | - | 25/15 | 50/85 | 14/10 | • 392  • 700 | Baslam et al., 2013 | 1 |
|  | Aragón | *Sm* 102F78 | GH | 2L, 4/pot | - | 25/15 | 50/85 | 14/10 | • 392  • 700 | Baslam et al., 2012 | 2 |
|  | AC Caribou | *• Sm* A2  *• Sm* NRG34 | GC | 3L, 10/pot | 600 | 22/17 | 70 | 16/8 | • 400  • 800 | Bertrand et al., 2007 | 3 |
|  | Aragón | *• Sm* 102F78  *• Sm* 102F34  *• Sm* 1032GMI | GH, Pamplona, Spain | 13L, 20/pot | - | • amb  • +4°C | - | - | • 380  • 700 | Sanz-Saez et al., 2012a | 4 |
|  | Aragón | *Sm*102F78 | TGE, Salamanca, Spain | 13L, 20/pot | - | • amb  • +4°C | - | - | • 400  • 720 | Aranjuelo et al., 2005 | 5 |
|  | Aragón | *Sm*102F78 | GH | 2L, 4/pot | - | 25/15 | 50/85 | 14/10 | • 395  • 700 | Goiocoechea et al., 2014 | 6 |
|  | Aragón | *Sm*102F78 | TGE, Salamanca, Spain | 13L, 20 plants/  pot | - | • amb  • +4°C | - | - | • 375  • 700 | Erice et al., 2006a | 7 |
|  | Arc | ND^‡^ | GC | 3.5L, 1/pot | 600 | • 15  • 20  • 25  • 30 | >50 | 14/10 | • 362  • 717 | Ziska and Bunce, 1994 | 8 |
|  | Aragón | *Sm*102F78 | GC | 2L, 4/pot | 600 | 25/15 | 40 | 14/10 | • 400  • 700 | Sanz-Saez et al., 2010 | 9 |
| *Trifolium repens*  (white clover) | Milkanova | *Rl trifolii* (RBL 5020) | GC | 1L, 1/pot | 500 | 18/13 | 80/90 | 16/8 | • 345  • 592 | Zanetti et al., 1998 | 10 |
|  | Blanca | ND^‡^ | GH, unknown location | 13 cm deep | - | - | - | - | • 367  • 620 | Nijs et al., 1988 | 11 |
|  | Milkanova | ND^‡^ | FACE, Eschikon, Switzerland | field | - | - | - | - | • 345  • 592 (DT) | Hebeisen et al., 1997 | 12 |
|  | Karina | ND | OTC, unknown location | 15L | - | - | - | - | • 390  • 470  • 550  • 630  • 710 | Manderscheid et al., 1997 | 13 |
| *Trifolium subterraneum*  (subclover) | Mt Barker | ND | TGE, Canberra, Australia | field | - | • amb  • +3.4 | - | - | • 380  • 690 | Lilley et al., 2001a | 14 |
|  | Mt Barker | ND | TGE, Canberra, Australia | field | - | • amb  • + 3.4 | - | - | • 380  • 690 | Lilley et al., 2001b | 15 |
| *Trifolium alexandrinum*  (berseem) | Pusa Jayant | *Rt* | OTC, unknown location | field | - | - | - | - | • 360  • 600 (DT) | Pal et al., 2004 | 16 |
| *Onobrychis viciifolia*  (sainfoin) | ND | ND | GC | 10L, 3/pot | 400 | 18.5-25.5/14-18 | 58/75 | ND | • 365  • 700 (DT) | Zhou and Shangguan, 2009 | 17 |
| *Lotus corniculatus*  (birdsfoot trefoil) | ND | ND^‡^ | GC | 1.9L, 1/pot | 300 | 21 max, 16.5 mean | 54 | 16/8 | • 345  • 590 | Ferris et al., 1993 | 18 |
| *Anthyllis vulneraria*  (kidney vetch) | ND | ND^‡^ | GC | 1.9L, 1/pot | 300 | 21 max, 16.5 mean | 54 | 16/8 | • 345  • 590 | Ferris et al., 1993 | 19 |
| Grain legumes |  |  |  |  |  |  |  |  |  |  |  |
| *Lens culinaris* (lentil) | 6 cultivars | Nodulator (BASF) or NoduleN (New Edge) | FACE, Horsham, Australia | field | - | - | - | - | • 400  • 550 (DT) | Bourgault et al., 2017 | 20 |
|  | 2 cultivars | *Rl* WSM1455  (NoduleN) | FACE, Horsham, Australia | field | - | - | - | - | • 400  • 550 (DT) | Parvin et al., 2019 | 21 |
|  | 2 cultivars | *Rl* WSM1455 | FACE, Horsham, Australia | field | - | - | - | - | • 400  • 550 (DT) | Parvin et al., 2018 | 22 |
| **Species** | **Cultivar** | **Symbiont** | **Growth** | **Pot size** | **Light (µmol m^-2^ s^-1^** | **Temp (°C)**  **(D/N)** | **Hum**  **(%)** | **Phot (h) (D/N)** | **[CO_2_]**  **(ppm)** | **Ref** | **No.** |
| *Vicia faba*  (faba bean) | Fiesta | *Rl* WSM1455 | FACE, Horsham, Australia | field | - | - | - | - | • 400  • 550 (DT) | Parvin et al., 2019 | 23 |
|  | Minica | ND | FCE, Wageningen, Netherlands | field | - | - | - | - | • 350  • 750 | Dijkstra et al., 1993 | 24 |
| *Glycine max*  (soybean) | 4 cultivars | ND | OTC, Harbin, China | 9L, 2/pot | - | - | - | - | • 390  • 550 | Li et al., 2018 | 25 |
|  | 18 cultivars | ND | FACE, Champaign, IL | field | - | - | - | - | • 380  • 550 (DT) | Bishop et al., 2015 | 26 |
|  | 24 cultivars | ND | OTC, Harbin, China | 9L, 2/pot | - | - | - | - | • 390  • 550 | Li et al., 2019 | 27 |
|  | 3 cultivars | ND | FACE, Champaign, IL | field | - | - | - | - | • 370  • 550 (DT) | Morgan et al., 2005 | 28 |
|  | 12 cultivars | *Bj* Mamezou^‡^ | TGE,  Marioka, Japan | 8L, 1/pot | - | - | - | - | • 400  • 595 | Kumagai et al., 2015 | 29 |
|  | Bragg | ND | OTC, unknown location | field | - | - | - | - | • 349  • 421-946 | Rogers et al., 1986 | 30 |
|  | Williams | *Bj*  USDA110 no. 2357 | FACE  Champaign, IL | field | - | - | - | - | • 400  • 600 (DT) | Sanz-Saez et al., 2015 | 31 |
|  | 2 cultivars | ND | GC | ND | 1000 | • 26/16  • 45/35 | ND | ND | • 360  • 700 | Bellaloui et al., 2016 | 32 |
|  | 8 cultivars | ND^‡^ | OTC,  Harbin, China | 9L, 2/pot | - | - | - | - | • 390  • 550 | Li et al., 2017 | 33 |
|  | Zhonghuang35 | ND^‡^ | OTC, Taigu, China | 67L, 8/pot | - | - | 60-70 | - | • 400  • 600 | Wang et al., 2018 | 34 |
|  | 2 cultivars | ND | OTC, Sao Paulo, Brazil | 5L | - | • amb  • +4 | - | - | • 380  • 800 | Palacios et al., 2019 | 35 |
|  | Thorne | ND | FACE, Champaign, IL | field | - | • amb  • +2.7/3.4 | - | - | • 400  • 600 | Köhler et al., 2019 | 36 |
| *Vigna radiata*  (mung bean) | Zhonglv 1 | ND | FACE, Beijing, China | field | - | - | - | - | • 400  • 550 | Ji et al., 2015 | 37 |
|  | Wilczek | ND | OTC, Beltsville, MD | field | - | - | - | - | • 413  • 667 | Ziska et al., 2007 | 38 |
| *Pisum sativum*  (field pea) | 5 cultivars | Inoculaid, Group E (Becker Underwood) | FACE, Horsham, Australia | field | - | - | - | - | • 400  • 550 (DT) | Bourgault et al., 2016 | 39 |
|  | PBA Twilight | ND | FACE, Horsham, Australia | 70L | - | - | - | - | • 390  • 550 | Jin et al., 2019 | 40 |
| *Cicer arietinum*  (chickpea) | Pusa 1105 | *Rhizobium* inoculant | OTC, New Delhi, India | field | - | - | - | - | • 384  • 580 (DT) | Saha et al., 2015b | 41 |
|  | Pusa 1105 | *Rhizobium* inoculant | OTC, New Delhi, India | field | - | - | - | - | • 384  • 580 (DT) | Saha et al., 2015a | 42 |
|  | Pusa 1105 | *Rhizobium* inoculant | OTC, New Delhi, India | field | - | - | - | - | • 390  • 580 | Saha et al., 2013 | 43 |
|  | Pusa 1105 | *Mc* SP4 | OTC, New Delhi, India | ND | - | - | - | - | • 370  • 550 (DT) | Pal et al., 2008 | 44 |
| *Phaseolus vulgaris*  (French bean) | 2 cultivars | ND | OTC, Bangalore, India | field | - | - | - | - | • 380  • 440 (DT) | Srinivasa Rao et al., 2015 | 45 |
| (kidney bean) | Redkloud | ND^‡^ | GH, Ithaca, NY | 10L, 1/pot | - | • 26/15  • 35/21 | 60-95 | 14/10 | • 345  • 690 | Jifon and Wolfe, 2005 | 46 |
|  | Montcalm | *Rhizobium* ‘Nitrogen’ (Lipha Tech Inc.)^‡^ | FCE, Gainesville, FL | field | - | • 28/18  • 34/24  • 40/30 | - | - | • 350  • 700 (DT) | Prasad et al., 2002 | 47 |
| *Cajanus cajan*  (pigeon pea) | ICPL 15011 | ND | OTC, Hyderabad, India | field | - | - | - | - | • 395  • 550 | Sreeharsha et al., 2015 | 48 |
| *C. cajan* | 3 cultivars | ND | OTC, unknown location | field | - | - | - | - | • 390  • 550 | Vanaja et al., 2015 | 49 |
| Tree legumes |  |  |  |  |  |  |  |  |  |  |  |
| *Acacia melanoxylon* | ND* | *B* sp. strain 29-24 | GC | 20L hyd tanks | 500 | 27/22 | ND | 14/10 | • 350  • 700 | Schortemeyer et al., 1999 | 50 |
| Other |  |  |  |  |  |  |  |  |  |  |  |
| *Arachis hypogaea*  (peanut) | Kadiri 3 | ND | GH, Nottingham, UK | field with liner at 1m | - | - | - | - | • 350  • 700 | Clifford et al., 1993 | 51 |
| *Medicago truncatula* | Jemalong  dnf1-1  dnf1-2 | *Sm* Rm1021 | OTC, Beijing, China | 27L, 1 plant/pot | - | - | - | - | • 390  • 750 | Guo et al., 2013a | 52 |
| *Medicago minima* | ND | *Sm* CM51 no4^‡^ | GH, Montpellier, France | 15.5L, 1/pot | - | - | - | - | • 370-395  • 700-750 | Roumet et al., 2000 | 53 |
| *Medicago glomerata* | ND | *Sm* CM41 no25^‡^ | GH, Montpellier, France | 15.5L, 1/pot | - | - | - | - | • 370-395  • 700-750 | Roumet et al., 2000 | 54 |
| *Lupinus perennis*  (wild lupine) | ND* | *Rhizobium* sp. (Prairie Moon Nursery) | GC | 10.5L, 1/pot | 600- 1100 | 25/20 | 60/80 | Mimic natural day length | • 365  • 700 | Lee et al., 2003 | 55 |

^†^ treated for > 3 weeks

^‡^ at least a subset of plants received additional N as fertilizer throughout experiment

Abbreviations: amb, ambient; *B*, *Bradyrhizobium*; Bj, *Bradyrhizobium japonicum*; DT, daytime only; FACE, free-air CO_2_ enrichment; FCE, field crop enclosure; GC, growth cabinet; GH, greenhouse; Hum, humidity; hyd, hydroponic; *Mc*, *Mesorhizobium cicer;* ND, no data; No., number assigned to study for use in tables; OTC, open top chamber; Phot, photoperiod; ppm, parts per million; *Rl*, *Rhizobium leguminosarum*; *Rt*, *Rhizobium trifolii*; *Sm*, *Sinorhizobium meliloti*; temp, temperature; TGE, temperature gradient enclosure.
